# Treatment planning comparison of focused very high energy electron and volumetric modulated arc therapy^☆^

**DOI:** 10.1016/j.phro.2026.100934

**Published:** 2026-02-20

**Authors:** Florian Amstutz, Chengchen Zhu, Werner Volken, Hannes A. Loebner, Silvan Mueller, Sascha Frei, Jenny Bertholet, Peter Manser, Michael K. Fix

**Affiliations:** Division of Medical Radiation Physics and Department of Radiation Oncology, Inselspital, Bern University Hospital, and University of Bern, Bern, Switzerland

**Keywords:** Very high energy electron radiotherapy (VHEE), Focused beams, Treatment planning, FLASH

## Abstract

**Background and purpose:**

Very high energy electron (VHEE) radiotherapy gained interest owing to technical advances and its potential for FLASH radiotherapy. Magnetically focused VHEE (fVHEE) beams showed promises in preliminary investigations in water phantoms. However, inverse treatment planning for fVHEE remains unexplored in clinically motivated scenarios. This study presented first inverse-optimized fVHEE treatment planning, permitting comparison to the current clinical benchmark, volumetric modulated arc therapy (VMAT), independent of FLASH considerations.

**Material and methods:**

Seven cases across five sites (brain, head and neck, lung, prostate, and femoral head) were investigated. fVHEE plans were generated using Monte Carlo-based beamlet dose calculations and in-house inverse optimization. Plans employed 250 MeV electrons focused via idealized magnetic lenses. Plan quality was compared regarding target coverage and organ-at-risk (OAR) sparing.

**Results:**

fVHEE achieved equivalent or improved target coverage in four sites, with V_95%_ increasing by up to 4.5% in lung cases. Notable OAR sparing occurred, including D_2%_ reductions of 0.2–7.6 Gy for the spinal canal and esophagus versus VMAT. The femoral head case’s D_2%_ to the rectum/bladder decreased by 1.3 Gy/2.6 Gy. Compared to VMAT, fVHEE enabled dose deposition via limited beam angles, reducing OAR dose at selected angles and to contralateral structures in lateralized tumors. Prostate cases showed less benefit due to target-OAR proximity in multiple directions, limiting directional selectivity.

**Conclusions:**

First results on inverse-optimized fVHEE planning are encouraging, particularly for lateralized targets or directionally isolated OARs. fVHEE demonstrates potential for selective dose sculpting, further comparison to VHEE is required to isolate fVHEE-specific benefits.

## Highlights

• First inverse-optimized treatment planning for focused very high energy electrons.
• Organ sparing and target coverage better than photon arc therapy in specific sites.
• With fewer beam angles, conformity was maintained and organ doses improved.
• Improved target coverage up to 4.5% and reduced organ near-max doses up to 7.6 Gy.
• Provides parameters and constraints relevant for future focused electron studies.

## Graphical abstract

**Figure:**
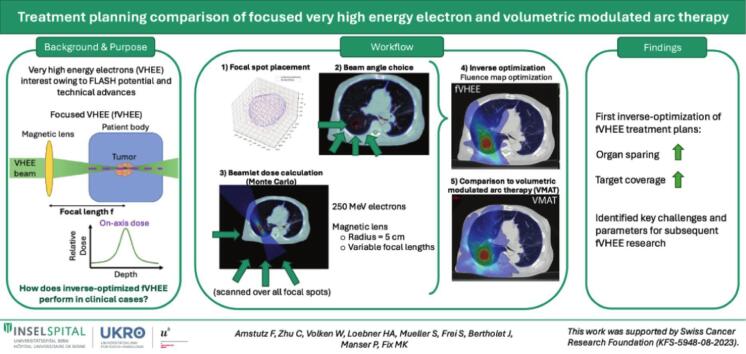

## 1 Introduction

Very high energy electron (VHEE) radiotherapy (RT) (50–250 MeV) was proposed two decades ago [1], [2], yet it has not reached clinics. Most patients receive (MV) photon RT, depositing dose along the entire beam path. Proton therapy offers a sharp distal fall-off but is sensitive to inhomogeneities and requires large and costly equipment. Clinical electron RT uses energy ranges of 4–22 MeV, delivered by standard linear accelerators, penetrating only approximately 10 cm with considerable lateral scattering, limiting use to superficial tumors or proposed as part of mixed-beam RT [3], [4], [5].

Recently, VHEE RT reemerged as a topic of interest, owing to the potential for FLASH dose rate and advances in accelerator technology. FLASH RT, originally demonstrated with few-MeV electrons (i.e. 4.5 MeV and 6 MeV) for superficial targets [6], motivate VHEE explorations, which may extend electron FLASH RT applications to deep-seated tumors [7]. Higher electron energies enable treating deep-seated tumors while reducing lateral scattering and showing less sensitivity to inhomogeneities compared to protons [8], [9], [10], [11], [12]. Moreover, VHEE machines could have smaller footprints than proton units [13], [14], [15], [16]. These advancements motivate identifying benefiting patient populations.

Another advantage of VHEE beams versus photons is their amenability to manipulation with magnetic fields. By employing magnetic lenses (e.g. quadrupoles) positioned in front of the patient, the electron beam can be focused [17], [18], [19], [20]. This focusing has potential to reduce entrance and exit doses, producing spread-out electron peaks analogous to proton spread-out Bragg peaks [19]. Early technical feasibility studies in research settings support these concepts [17], [18], [19], [20], though limited to measurements on simple geometric phantoms. Applications on clinically motivated cases with inverse planning are lacking.

Treatment planning for VHEE has only been addressed in a few studies, so far showing potential in clinically relevant scenarios [9], [12], [15], [21], [22], [23], [24], [25], [26]. Consequently, VHEE treatment planning does not yet match the sophistication of established RT modalities in aspects such as optimal beam energy selection, beam angle choice, and beam delivery system (scattering or scanning). In-depth treatment planning studies could help explore the clinical situations where VHEE is most promising and could inform design and development of treatment machines, several of which are in planning or construction stages [13], [27], [28] at different institutes [29], [30], [31]. While planning studies exist for VHEE [9], [12], [15], [21], [22], [23], [24], [25], [26], similar investigations are lacking for magnetically focused VHEE (fVHEE).

This study presents the first inverse treatment planning for fVHEE, providing insights into achievable treatment plan quality with fVHEE in clinical cases, without considering FLASH. We investigated a range of indications to assess potential benefits and identified challenges and open questions. Seven clinically motivated cases across five treatment sites were investigated and compared to photon RT delivered via volumetric modulated arc therapy (VMAT).

## 2 Materials and methods

### 2.1 fVHEE beamlet dose calculation

Dose calculation used an in-house tool based on Swiss Monte Carlo Plan (SMCP) [32], enabling fVHEE beamlet dose calculation. The planning workflow comprised two main stages: (1) calculation of fVHEE beamlet doses scanned over the target from all beam angles and for each field covering the target in beams-eye view plus a margin and (2) inverse optimization of beamlet weights to meet user specified objectives. The workflow is shown in Supplementary Fig. S1.

For beamlet dose calculation, the first step entailed providing all beamlet parameters (Table 1). This includes giving the planning target volume (PTV) and body contour. Defining a margin (m) representing a uniform expansion around the PTV for placement of additional beamlets for better conformity. A spacing factor (k) defines the distance factor for adjacent focal spots in the diamond pattern (described below). For each case, beam angles were defined manually with the intention to omit critical structures (Table 1) with the treatment table fixed at 0°. The electron beam energy was fixed to 250 MeV for all beamlets.

**Table 1:** Case description and beam/beamlet setup for all cases.

Patient Case	PTV Volume [cm^3^]	Dose Prescription and Fractionation	PTV Normalization	fVHEE Beam Angles [°]	fVHEE Beamlet Grid Points	fVHEE Beam Energy [MeV]	fVHEE Margin (m) [cm]	fVHEE Spacing factor (k) [cm]	VMAT Beam Energy [MV]	VMAT Arc Configuration (*CW: clockwise. CCW: counterclockwise. CA: collimator angle. Treatment table at 0° if not stated differently)*
Brain (B)	43.9	2 × 6 Gy Total: 12 Gy	D_95%_ = 100% D_pres_	−130, −90, 130	1635	250	0.5	0.3	6	[179° CCW 181°, CA 355°], [181° CW 179°, CA 5°]
Head and neck (H)	63.7	25 × 2 Gy Total: 50 Gy	D_95%_ = 100% D_pres_	−20, 0, 30, 90	3120	250	0.5	0.3	6	[136° CCW 222°, CA 85°], [222° CW 136°, CA 5°], [355° CW 55°, CA 88°, table at 90°]
Lung 1 (L1)	26.9	30 × 2 Gy Total: 60 Gy	D_50%_ = 100% D_pres_	−150, −90, 150, 180	1284	250	0.5	0.3	6	[181° CW 20°, CA 2°], [20° CCW 181°, CA 88°]
Lung 2 (L2)	37.4	30 × 2 Gy Total: 60 Gy	D_50%_ = 100% D_pres_	−130, −90, 130, 180	1932	250	0.5	0.3	6	[181° CW 179°, CA 5°], [179° CCW 181°, CA 85°]
Prostate 1 (P1)	119.5	30 × 2 Gy Total: 60 Gy	D_50%_ = 100% D_pres_	−150, −110, −20, 20, 110, 150	2649	250	0.5	0.4	6	[181° CW 179°, CA 2°], [179° CCW 181°, CA 88°]
Prostate 2 (P2)	62.2	30 × 2 Gy Total: 60 Gy	D_50%_ = 100% D_pres_	−150, −110, −20, 20, 110, 150	1578	250	0.5	0.4	6	[181° CW 179°, CA 5°], [179° CCW 181°, CA 85°]
Femoral Head (F)	145.9	10 × 4.65 Gy Total: 46.5 Gy	D_50%_ = 100% D_pres_	−25, 0, 25, 90, 180	2930	250	0.25	0.4	6	[179° CCW 310°, CA 358°], [310° CW 179°, CA 2°], [179° CCW 310°, CA 92°], [310° CW 179°, CA 88°]

Several key assumptions were made, as clinical systems are unavailable. Technical feasibility of the presented fVHEE requires proof. Beamlets were assumed to be symmetrically focused in x and y directions by the lens geometry in a purely geometrical model without explicit electromagnetic simulation of the lens. In contrast, real quadrupole lenses focus in one transverse direction while simultaneously defocusing in the other. Combining a quadrupole sequence achieves a net focusing effect [18], [19], [20]. This intrinsic asymmetry of quadrupoles may introduce additional challenges or be exploited advantageously. Each beamlet exhibited the prescribed geometric focusing down to its nominal full width half maximum (FWHM) at the focal spot, while the actual FWHM increased in tissue due to scattering. The practicability of dynamically adapting focal length via magnetic lenses needs to be tested [19], [33]. Focusing beamlet parameters provided by the user were: A) Magnetic lens radius fixed at 5 cm (defines geometric focusing together with geometric focal spot width at FWHM). This value was motivated by previous investigations in water tanks [20]. B) Focal spot’s geometric FWHM at isocenter was set to 1 cm (motivated by [19]).

**Fig. 1:**
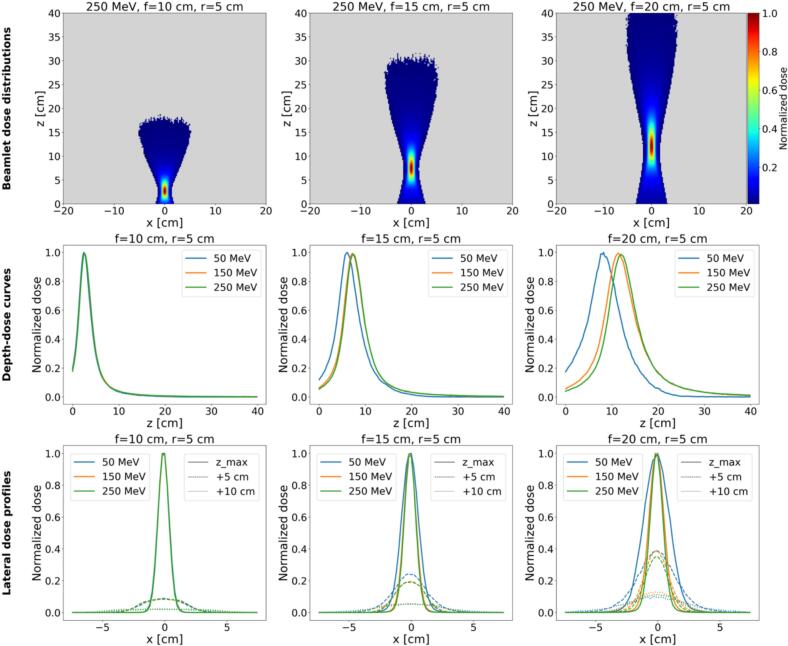
The top row shows dose distributions of an idealized 250 MeV fVHEE beam with a magnetic lens placed 7 cm before the water box, with a lens radius of 5 cm and different focal lengths (10 cm, 15 cm, 20 cm). The middle row shows for the same geometries the depth-dose curves at the central axis for different energies (50 MeV, 150 MeV, 250 MeV). The bottom row shows the according lateral profiles along x at the dose maximum depth, 5 cm, and 10 cm, behind the according dose maximum.

Beam characterization examples in water, including dose distributions, depth-dose curves and lateral profiles illustrating geometric focusing are shown in Fig. 1 (additional details in Supplementary Figs. S2–S4).

To cover the PTV plus margin, the focused beamlets were scanned (step-and-shoot) over the target by changing those beamlets’ position and the focal length of the magnetic lens. This scanning was performed from each of the defined beam angles. The beamlet position could be changed either by scanning or by moving the treatment table, as applied for proton therapy [34]. These grid points for the foci of the fVHEE beamlets were defined once for all the beam directions. The spots were arranged in a 3D grid following a diamond-like pattern (Supplementary Fig. S1), covering the PTV plus m. The grid spacing was defined over k, ensuring that the grid conformed to the PTV shape. The detailed spot placement is explained in the Supplementary Material A.

Spot placement did not mirror a commercial treatment planning system, but the hexagonal lattice was motivated by the 2D distribution per energy layer for proton therapy [35], to achieve compact, quasi-uniform PTV coverage.

For each retained spot and beam direction, the fVHEE beamlets were “scanned” by shifting the focal position. For this study, all beamlets were simulated with an energy of 250 MeV, which probably represented an upper-limit and best-case scenario, as preliminary investigations showed improved robustness of the focused beams against longer focal lengths and tissue inhomogeneities for energies above 200 MeV [36] (Supplementary Figs. S2-S4). Dose distributions for all beamlets were computed using EGSnrc Monte Carlo code [37], where focusing was modeled purely by the geometric lens parameters defined above. Simulating 10^6^ particle histories per beamlet kept mean statistical uncertainty <1% for voxels exceeding 50% of the maximum dose.

### 2.2 Treatment plan optimization and evaluation

For optimizations, we used our validated in-house developed inverse optimizer [4], [5], [38], [39], [40], [41], [42], [43], optimizing objective functions according to dose objectives via fluence map optimization. As input, we provided the calculated beamlets, and as output, the beamlet weights were determined. To ensure robust evaluation, fVHEE was compared against two distinct VMAT references: A) An “optimizer-matched” VMAT generated using the same in-house inverse optimizer and dose objectives as the fVHEE plans. This optimizer-matched VMAT served as a control to isolate modality effects without confounding from different optimizers or objective functions. Dose objectives, specific objectives and objective function definition [40] are provided in the Supplementary Material B Tables S8–S10. B) The clinical VMAT optimized in the Eclipse treatment planning system (Siemens Healthineers, Erlangen, Germany) represented the original clinical plan. This serves as the “plan to beat” assessing whether fVHEE could meet current clinical standards. VMAT plans used 6 MV photons, with dose calculated using Analytical Anisotropic Algorithm (AAA).

Optimized doses were imported into the Eclipse research system for visual plan evaluation. Furthermore, dose volume histograms (DVHs) as well as dose parameters, homogeneity index () and Paddick conformity index (CI) were compared across modalities. The RATING score according to the filled table in the Supplementary Material C Table S11 was 86% [44].

### 2.3 Patient cases

**Fig. 2:**
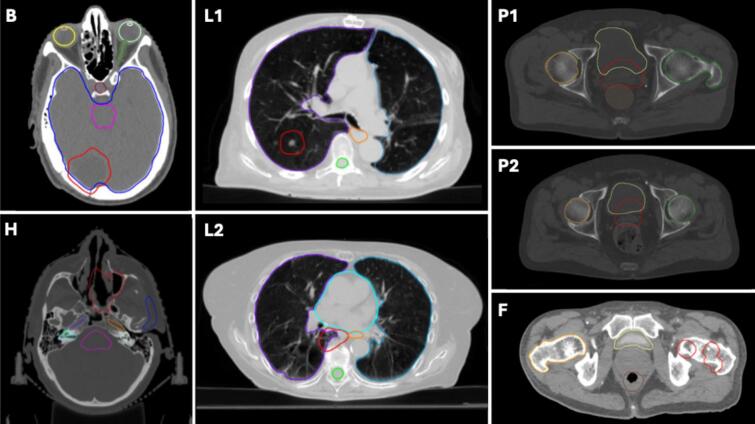
Patient anatomies showing the PTV (red) and OARs for the seven cases. Case “Brain” (B) includes the brain (blue), brainstem (pink), left eye (light green), right eye (yellow), left lens (white), right lens (orange), optic nerve (dark green), and pituitary gland (bordeaux). Case “Head and Neck” (H) includes the brainstem (pink), parotid gland (blue), left inner ear (light green), right inner ear (cyan), left cochlea (grey), right cochlea (grass green), left carotid artery (orange), and right carotid artery (violet). Case “Lung 1” (L1) includes the left lung (marine blue), right lung (violet), spinal canal (green) and esophagus (orange), while Case “Lung 2” (L2) includes the left lung (marine blue), right lung (violet), spinal canal (green), esophagus (orange), and heart (cyan). Case “Femoral Head” (F) includes the bladder (yellow), rectum (brown), and right femoral head (orange). Cases “Prostate 1” (P1) and “Prostate 2” (P2) include the bladder (yellow), rectum (brown), left femoral head (green), and right femoral head (orange). (For interpretation of the references to colour in this figure legend, the reader is referred to the web version of this article.)

Seven anonymized, retrospectively selected cases were investigated to represent a range of treatment indications, including the brain (B), head and neck (H), lung (L), prostate (P), and femoral head (F). Ethical approval was not required for this study as it utilized exclusively anonymized, non-linkable patient datasets for quality assurance of a non-existent treatment modality. The brain case had a planning target volume (PTV) located in the posterior brain. The PTV in the H case was in the nasal cavity. The two lung cancer cases involved one PTV located peripherally in the right upper lobe, and one centrally located adjacent to the heart and esophagus, with proximity to the thoracic spine. The two prostate cases were both cases without PTV extensions to the vesicles. The femoral head case included a PTV composed of two subparts located in the left leg. The tumor locations can be seen in Fig. 2.

PTV volumes, prescription doses, and fractionation schemes for all cases are summarized in Table 1. For this study, the prescription doses were based on the clinically created VMAT plans. As this was an early-phase investigation, the primary goal was to compare fVHEE treatment plans to the corresponding existing clinical VMAT plans.

## 3 Results

**Fig. 3:**
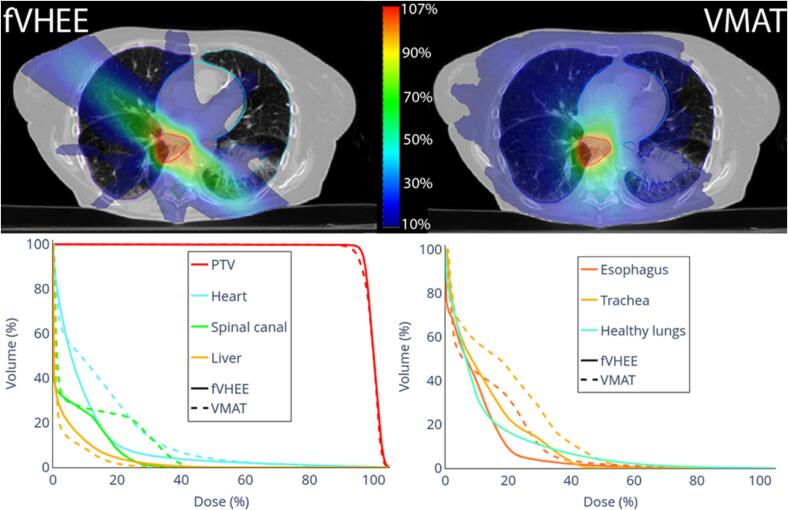
Dose distributions of fVHEE and the clinical VMAT in a representative transversal slice with DVHs for lung cancer case L2.

In lung cases, fVHEE matched or slightly improved PTV coverage versus clinical VMAT (V_95%_: +0.2% and +4.5%, Table 2). Improved the near-maximum dose for the esophagus and the spinal canal, with D_2%_ reductions between 0.2 and 7.6 Gy. For L1 (Supplementary Fig. S5), D_mean_ to the heart and V_20Gy_ to the healthy lungs (lungs-PTV) were similar between fVHEE and clinical VMAT. For L2 (Fig. 3), heart D_mean_ was 2.7 Gy lower, while D_2%_ to the liver and V_20Gy_ for the healthy lungs increased from 11.5 Gy to 16.9 Gy and from 4.1% to 9.2% compared to clinical VMAT, respectively. Overall, DVHs showed especially for L2, improvements for most OARs. In both cases, OARs like the spinal canal and the esophagus could be spared by the possibility of delivering the dose from a limited number of beam angles, while keeping target coverage and conformity. The cost was broader intermediate-dose spread along the beam path that passed between the heart, spinal canal and esophagus (Fig. 3).

**Table 2:** DVH parameters for fVHEE and VMAT plans and their differences in Gy or % points. For each metric, the best value is shown in **bold**.

Targets/OARs	Param.	fVHEE	clinical VMAT	Diff.		Targets/OARs	Param.	fVHEE	clinical VMAT	Diff.
**Brain**						**Head and neck**				
PTV	D_2%_ [Gy]	**13.0**	13.8	−0.8		PTV	D_2%_ [Gy]	54.9	**54.7**	0.2
	V_95%_ [%]	99.5	**99.7**	−0.2			V_95%_ [%]	98.7	**99.1**	−0.4
	HI [–]	**0.09**	0.16	−0.07			HI [–]	**0.10**	0.11	−0.01
	CI [–]	**0.92**	0.89	0.03			CI [–]	0.45	**0.47**	−0.02
Brain	D_mean_ [Gy]	**1.6**	1.7	−0.1		Brainstem	D_2%_ [Gy]	**16.0**	19.7	−3.7
Brainstem	D_2%_ [Gy]	0.9	**0.8**	0.2		Cochlea L	D_mean_ [Gy]	**8.1**	10.5	−2.4
Normal Tissue	D_mean_ [Gy]	**0.6**	**0.6**	0.0		Cochlea R	D_mean_ [Gy]	**6.2**	8.8	−2.6
**Lung 1**						Eye L	D_2%_ [Gy]	**32.4**	33.0	−0.7
PTV	D2% [Gy]	61.6	**61.2**	0.4		Eye R	D_2%_ [Gy]	25.8	**19.6**	6.2
	V95% [%]	98.9	**98.7**	0.2		Chiasm	D_2%_ [Gy]	**29.5**	32.7	−3.2
	HI [–]	**0.05**	**0.05**	0.00		Oral cavity	D_mean_ [Gy]	5.9	**5.4**	0.5
	CI [–]	**0.47**	0.45	0.01		Optic nerve L	D_2%_ [Gy]	**40.0**	45.4	−5.3
Heart	D_mean_ [Gy]	0.4	**0.3**	0.1		Optic nerve R	D_2%_ [Gy]	**24.4**	26.1	−1.8
Spinal Canal	D_2%_ [Gy]	**4.6**	11.9	−7.3		Carotid L	D_mean_ [Gy]	**9.2**	15.9	−6.7
Esophagus	D_2%_ [Gy]	5.1	**4.9**	0.2		Carotid R	D_mean_ [Gy]	6.5	**6.6**	−0.2
Lungs-PTV	V_20Gy_ [%]	**4.8**	5.1	−0.3		Spinal Canal	D_2%_ [Gy]	**6.7**	11.4	−4.7
Normal Tissue	D_mean_ [Gy]	**1.3**	1.5	−0.1		Parotid L	D_mean_ [Gy]	**2.3**	3.4	−1.2
**Lung 2**						Parotid R	D_mean_ [Gy]	2.3	**1.6**	0.7
PTV	D_2%_ [Gy]	62.6	**62.4**	0.2		Lacrymal Gland L	D_mean_ [Gy]	7.9	**5.2**	2.7
	V_95%_ [%]	**97.7**	93.4	4.4		Lacrymal Gland R	D_mean_ [Gy]	11.4	**5.2**	6.2
	HI [–]	**0.08**	0.10	−0.02		Lips	D_mean_ [Gy]	4.1	**3.3**	0.8
	CI [–]	0.41	**0.47**	−0.06		Normal Tissue	D_mean_ [Gy]	4.4	**4.3**	0.0
Heart	D_mean_ [Gy]	**5.9**	8.5	−2.7		**Prostate 1**				
Spinal Canal	D_2%_ [Gy]	**16.2**	23.6	−7.4		PTV	D_2%_ [Gy]	62.4	**61.7**	0.7
Esophagus	D_2%_ [Gy]	**23.8**	29.1	−5.3			V_95%_ [%]	97.3	**99.1**	−1.7
Trachea	D_mean_ [Gy]	**7.7**	11.3	−3.6			HI [–]	0.11	**0.06**	0.05
Liver	D_mean_ [Gy]	2.0	**1.5**	0.5			CI [–]	0.44	**0.48**	−0.04
Lungs-PTV	D_mean_ [Gy]	**6.9**	7.1	−0.2		Bladder healthy	D_mean_ [Gy]	12.7	**11.6**	1.1
	V_20Gy_ [%]	9.2	4.1	5.1		Rectum healthy	D_2%_ [Gy]	52.1	**49.1**	3.1
Normal Tissue	D_mean_ [Gy]	**2.5**	2.6	−0.2			D_mean_ [Gy]	11.2	**9.7**	1.5
**Femoral Head**						Femoral Head L	D_mean_ [Gy]	**6.6**	9.7	−3.1
PTV	D_2%_ [Gy]	**48.3**	**48.3**	0.0		Femoral Head R	mean [Gy]	**8.1**	11.6	−3.5
	V_95%_ [%]	**96.4**	96.1	0.3		Bowel	D_2%_ [Gy]	**1.5**	2.6	−1.1
	HI [–]	**0.09**	**0.09**	0.00		Penile bulp	mean [Gy]	10.6	**6.8**	3.7
	CI [–]	**0.48**	**0.48**	0.00		Normal Tissue	mean [Gy]	**3.2**	3.4	−0.2
Bladder	D_2%_ [Gy]	**0.9**	3.6	−2.6		**Prostate 2**				
	D_mean_ [Gy]	**0.5**	1.7	−1.2		PTV	D_2%_ [Gy]	62.5	**62.1**	0.4
Rectum	D_2%_ [Gy]	**1.1**	2.4	−1.3			V_95%_ [%]	96.5	**98.3**	−1.9
	D_mean_ [Gy]	**0.5**	1.3	−0.8			HI [–]	0.09	**0.07**	0.02
Femoral Head R	D_mean_ [Gy]	**0.3**	0.7	−0.4			CI [–]	**0.48**	0.47	0.01
Normal Tissue	D_mean_ [Gy]	**2.2**	2.6	−0.4		Bladder healthy	D_mean_ [Gy]	29.3	**26.8**	2.5
						Rectum healthy	D_2%_ [Gy]	53.4	**52.0**	1.4
							D_mean_ [Gy]	18.0	**16.1**	1.9
						Femoral Head L	D_mean_ [Gy]	**5.1**	5.2	−0.1
						Femoral Head R	D_mean_ [Gy]	5.2	**4.7**	0.5
						Bowel	D_2%_ [Gy]	8.6	**3.9**	4.7
						Normal Tissue	D_mean_ [Gy]	**1.9**	2.0	−0.1

**Fig. 4:**
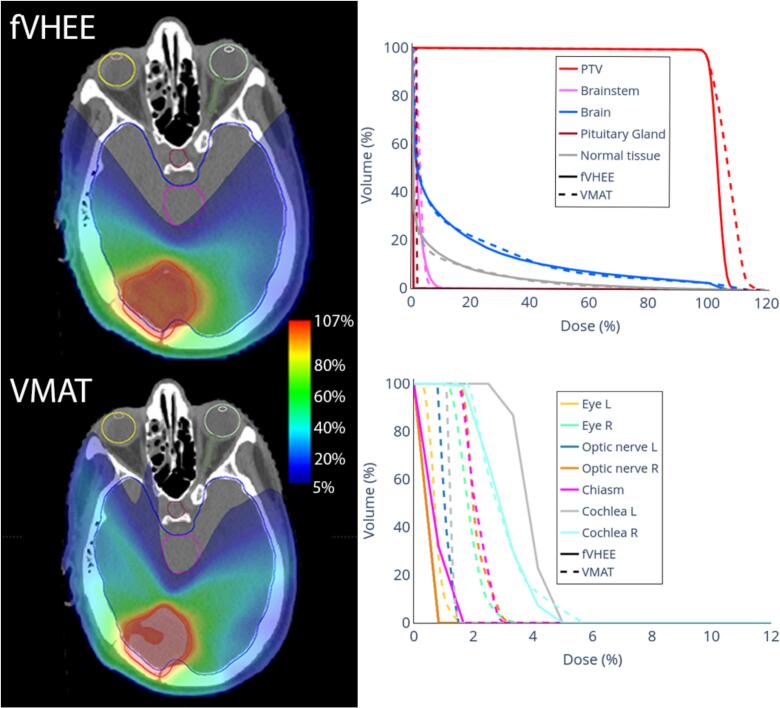
Dose distributions of fVHEE and the clinical VMAT in a representative transversal slice with DVHs for the brain case B.

Case B (Fig. 4) showed similar target coverage and OAR doses versus clinical VMAT. Dose to the optic system, located in the low dose regions, was lower for fVHEE compared to clinical VMAT. For case B, fVHEE achieved a D_2%_ in the PTV of 13.0 Gy, which was close to the 12.9 Gy achieved by the optimizer-matched VMAT. The higher hotspot observed in the clinical VMAT (13.8 Gy) suggests that this observed difference is driven by optimization and planning intent, highlighting the dependence on comparator choice. In case H (Supplementary Fig. S6), fVHEE improved target coverage, while doses to most OARs were reduced. But for the oral cavity and right parotid D_mean_ increased by 0.5 Gy and 0.7 Gy. Additionally, the dose to the right eye and right lacrimal gland increased. The brainstem, chiasm, left parotid, and spinal canal all showed reduced near-maximum and mean doses (Table 2 and Supplementary Table S2).

For prostate cases, fVHEE in the current setting showed worse target coverage, increased doses to several OARs, and only minor OAR improvements in a few instances. Corresponding dose distributions and DVHs are in the Supplementary Figs. S7 and S8.

For the unilateral femoral head case (Supplementary Fig. S9), fVHEE showed improvement for all OARs, with a similar PTV coverage (D_2%_: +0 Gy, V_95%_: +0.3%) compared to clinical VMAT. Near-maximum doses were reduced for the rectum (1.3 Gy), bladder (2.6 Gy), and the contralateral femoral head (1.0 Gy). Furthermore, mean doses to these OARs and the healthy tissue were reduced between 0.4 and 1.2 Gy. For fVHEE, the dose could be contained to the ipsilateral side, while central parts with the rectum and the bladder were mostly spared from any dose, and the contralateral side was spared completely.

The DVH parameters of optimizer-matched VMAT plans and the respective differences to fVHEE and the clinical VMAT are summarized in the Supplementary Tables S1–S7.

## 4 Discussion

This study presented the first investigation of inversely optimized treatment planning for fVHEE across multiple cases and anatomical sites. The results demonstrated that fVHEE has the potential to achieve high-quality treatment plans with similar or superior performance to clinical VMAT in selected indications, not including any FLASH considerations.

Our findings suggest that fVHEE offers particular benefits compared to VMAT in anatomies when tumors are located laterally or when only part of the beam direction space is restricted by OARs. For instance, in the L and F cases, fVHEE achieved similar or improved target coverage with reductions in OAR doses. Our fVHEE planning framework achieved these improvements compared to VMAT, with fVHEE’s ability to deliver the dose more directionally, which more selectively spared OARs. Intensity-modulated radiation therapy (IMRT) or partial arcs can also restrict directions, but they do not necessarily achieve the same overall plan quality. Future comparisons to other photon modalities will be needed.

In the B and H case, dose conformity of our fVHEE framework increased compared to VMAT, with sparing of many OARs, especially OARs located at a distance to PTVs. This distant sparing likely stems from fVHEE beam directionality, which enables trajectories that completely avoid specific structures. However, isolating this from fVHEE-specific depth-dose effects would require further comparison with other directional modalities like IMRT. fVHEE was less successful in prostate cases. Due to the bladder and the rectum partly overlapping with the PTV in the anterior, respectively posterior direction, the directional freedom for the fVHEE beams is limited. Thus, under current assumptions, prostate treatments may be challenging, and this patient group may not benefit. PTV-OAR overlap occurs in other sites, potentially limiting fVHEE beyond prostate. Improved spot placement or multi-energy layering could be investigated as mitigation option. However, for the prostate cases it was mostly due to two OARs constraining opposing directions. Furthermore, diverging VHEE could be an option for prostate cases [15].

The unilateral femoral head case illustrated an advantageous scenario, by restricting dose deposition to one side of the body while sparing contralateral femoral head, bladder, and rectum shows potential for treating localized extremity tumors with minimal surrounding irradiation. Additionally, such cases may represent ideal initial clinical indications for evaluating the safety and feasibility of fVHEE in FLASH radiotherapy, analogous to the early proton FLASH investigations [45].

All results were evaluated without considering any biological enhancement owing to the FLASH effect or spatial fractionation (SFRT). Should future advances allow FLASH delivery with fVHEE, the observed benefits could be amplified, and additional benefits could appear. For example, normal tissue volumes receiving intermediate dose that are currently considered suboptimal may become acceptable or even advantageous if they trigger a protective FLASH response in normal tissue. As such, cases like prostate could benefit from fVHEE as in combination with FLASH the structures close to the target might be saved better. However, this remains speculative and will depend on a precise understanding of dose and dose-rate thresholds (if they exist), temporal beam characteristics, and tissue-specific responses. This is particularly relevant for scanned delivery, where magnet refocusing and inter-spot switching times warrant careful future consideration. Alternatively, fVHEE could be used to deliver SFRT treatments for an improved therapeutic outcome [46].

This work should be considered an initial exploration of the potential of fVHEE and a first source of insight to guide future research by identifying challenges that come with actual patient cases and show planning-level parameters to be further investigated, which then down the road would help define technical requirements of machines. While beam focusing with realistic magnetic lenses will need further investigations, we assumed in this work that a lens radius, respectively a beam pipe radius, of 5 cm is technically feasible. The impact of lens size warrants further investigation, as variations in radius may influence both the achievable focusing strength and, consequently, treatment plan quality. In addition, this work used a fixed 250 MeV beam energy to ensure depth coverage for all cases and to test a robust focusing condition. The impact on plan quality with lower energies needs further considerations. The use of multiple beam energies could enable finer modulation of dose deposition at different depths and thereby enhance plan conformity, in analogy to energy-layered delivery in proton therapy. However, fVHEE at fixed energies could present an important FLASH delivery benefit compared to proton therapy. By shifting the dose maximum in depth, it could preserve good conformity, without the need of energy switching, which is relatively slow.

We assumed ideal scanning of focal spots, by beam steering or table movement. This method needs to be technically validated, particularly in terms of mechanical accuracy, timing constraints, and potential patient motion. Relatedly, robustness analyses will be essential to evaluate dose distributions’ sensitivity to uncertainties. Potential interplay effects in moving targets must be investigated, drawing on lessons from proton therapy [47]. Furthermore, alternative spot placement patterns, individual spot placement per beam, or adaptive placements could be investigated. This was a conceptual, first-step study under idealized beam focusing: future work should compare fVHEE to unfocused/divergent VHEE (and to proton therapy where relevant) and explore more realistic beam-optics models to assess when adding magnetic focusing justifies the extra complexity.

Radiosurgery, oligometastatic or multifocal disease represent future directions for fVHEE research. The beam steering capabilities and rapid fall-off outside the focus region may enable efficient delivery of ablative doses to small lesions with minimal normal tissue exposure.

In conclusion, this study has successfully implemented and tested an inverse optimized treatment planning framework for fVHEE. Our results suggest that fVHEE has, at this conceptual stage, potential for selected indications, particularly in anatomies where effective treatment can be achieved by selecting beam directions that avoid OARs, unlike VMAT which requires irradiation from many directions. The observed differences cannot be attributed solely to focusing physics and comparisons to unfocused VHEE will be important to evaluate the necessity of added technical complexity. While technical, treatment planning, and biological challenges remain, these findings provide an important foundation for future exploration of fVHEE, both as standalone modality and as potential enabler for deep-seated FLASH radiotherapy.

**Support:** This work was supported by 10.13039/501100013362Swiss Cancer Research Foundation (KFS-5948-08-2023).

## Declaration of Generative AI and AI-assisted technologies in the writing process

During the preparation of this work the author used chatGPT to improve readability and language. After using this tool/service, the authors reviewed and edited the content as needed and take full responsibility for the content of the publication.

## CRediT authorship contribution statement

**Florian Amstutz:** Writing – original draft, Visualization, Software, Project administration, Methodology, Investigation, Funding acquisition, Data curation, Conceptualization. **Chengchen Zhu:** Writing – review & editing, Software, Investigation, Data curation. **Werner Volken:** Writing – review & editing, Software, Data curation. **Hannes A. Loebner:** Writing – review & editing, Software. **Silvan Mueller:** Writing – review & editing, Software, Data curation. **Sascha Frei:** Writing – review & editing, Software, Methodology, Investigation. **Jenny Bertholet:** Writing – review & editing, Software, Funding acquisition, Data curation. **Peter Manser:** Writing – review & editing, Supervision, Project administration, Funding acquisition, Conceptualization. **Michael K. Fix:** Writing – review & editing, Supervision, Project administration, Funding acquisition, Conceptualization.

## Declaration of competing interest

The authors declare that they have no known competing financial interests or personal relationships that could have appeared to influence the work reported in this paper.
