# Supplementary material for: Treatment planning comparison of focused very high energy electron and volumetric modulated arc therapy
Source: Phys Imaging Radiat Oncol. 2026 Feb 20;37:100934. doi: 10.1016/j.phro.2026.100934 (PMC12955155; doi:10.1016/j.phro.2026.100934)
Supplement: Supplementary Data 1 [file mmc1.pdf]

Supplementary material: Treatment planning comparison of focused very high energy electron and volumetric modulated arc therapy

Florian Amstutz, Chengchen Zhu, Werner Volken, Hannes A Loebner, Silvan Mueller, Sascha Frei, Jenny Bertholet, Peter Manser, Michael K Fix

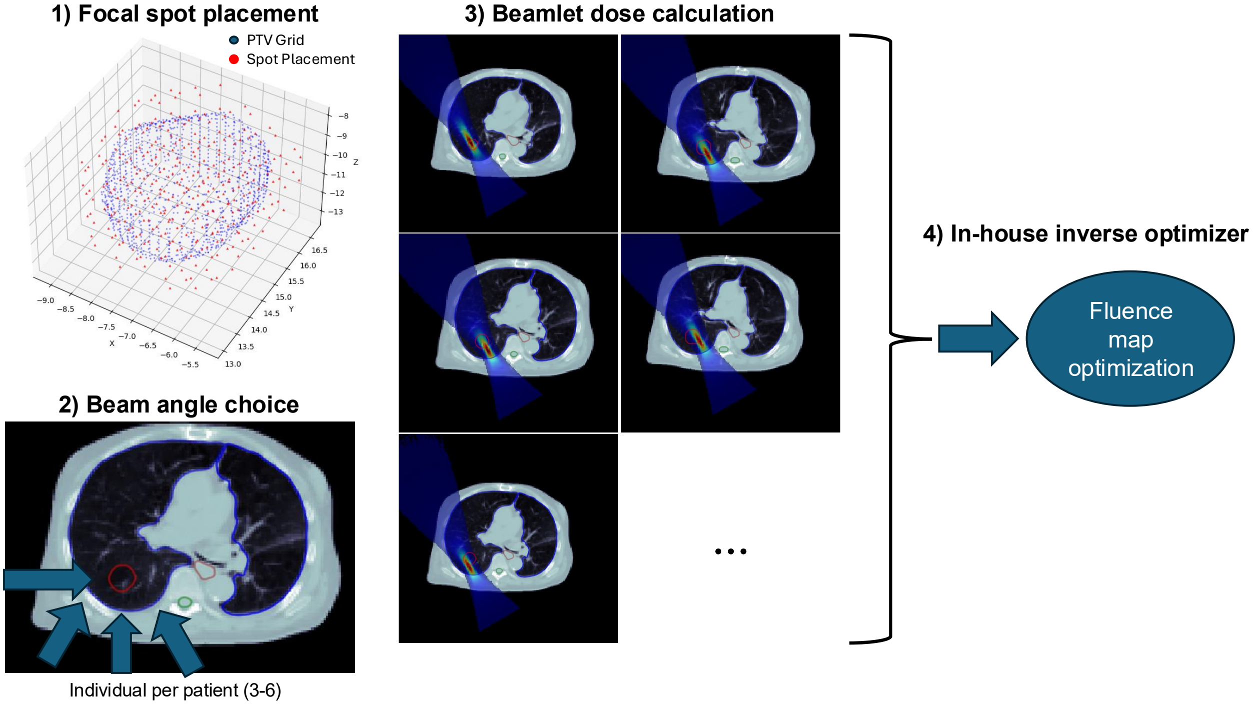

Figure S1: Schematic workflow of the treatment planning process.

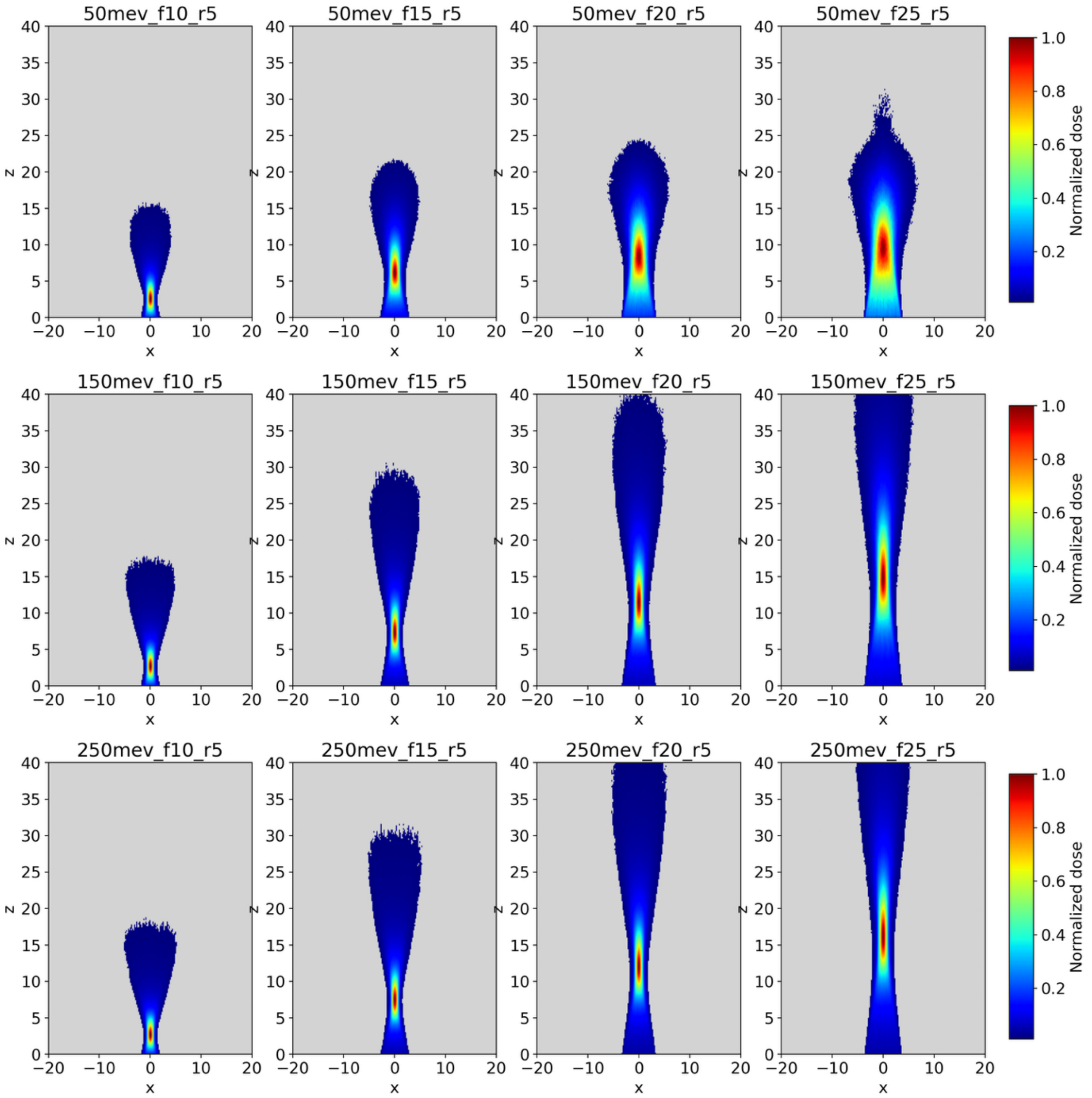

Figure S2: Dose distributions of an idealized fHEE beam with a magnetic lens placed 7 cm before the water box, for different energies 50 MeV (top row), 150 MeV (middle row), and 250 MeV (bottom row) and different focal lengths 10-25 cm (f10, f15, f20, f25) and a lens radius of 5 cm (r5).

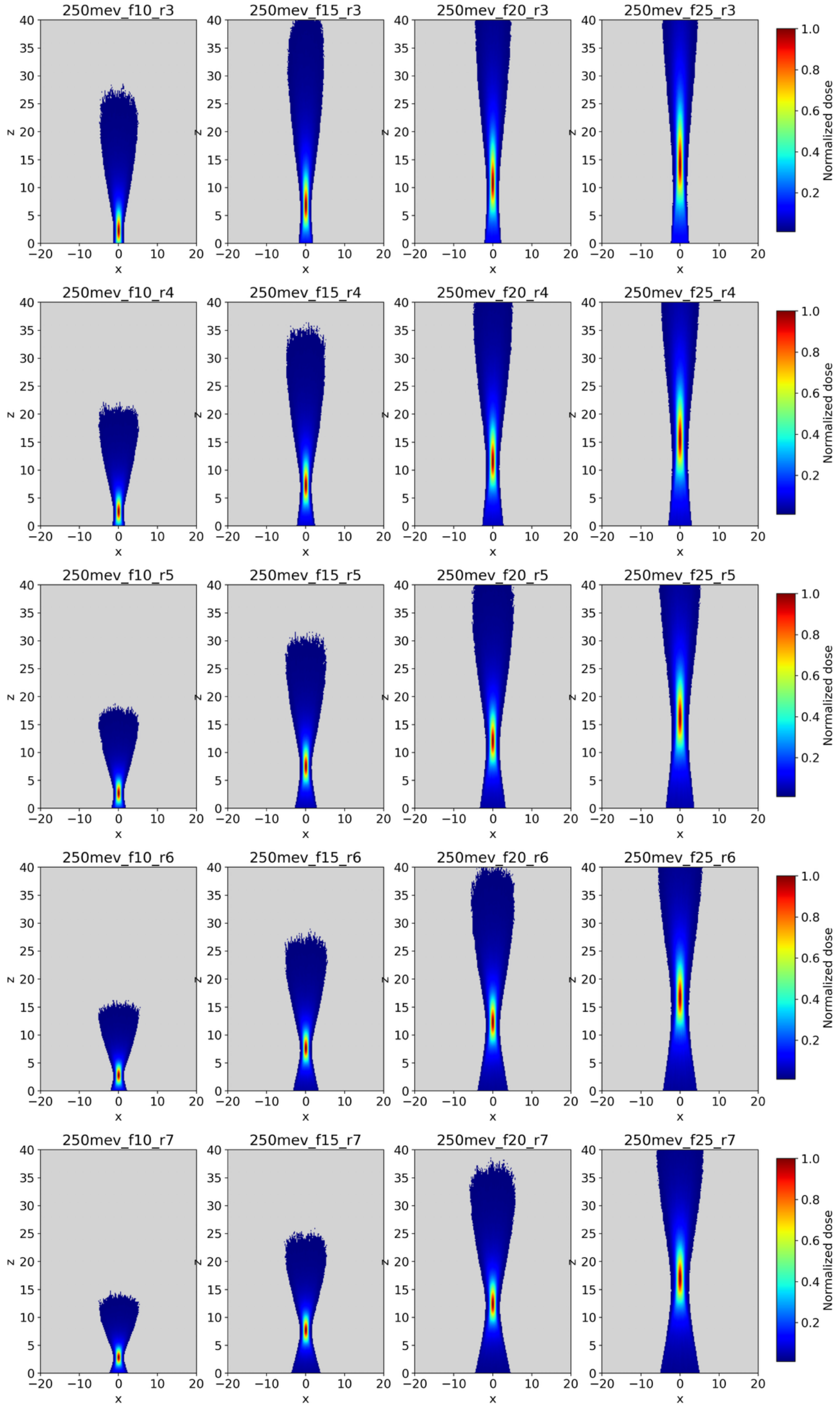

Figure S3: Dose distributions of an idealized 250 MeV fHEE beam with a magnetic lens placed 7 cm before the water box, for different lens radii 3-7 cm (r3, r4, r5, r6, r7) and different focal lengths 10-25 cm (f10, f15, f20, f25).

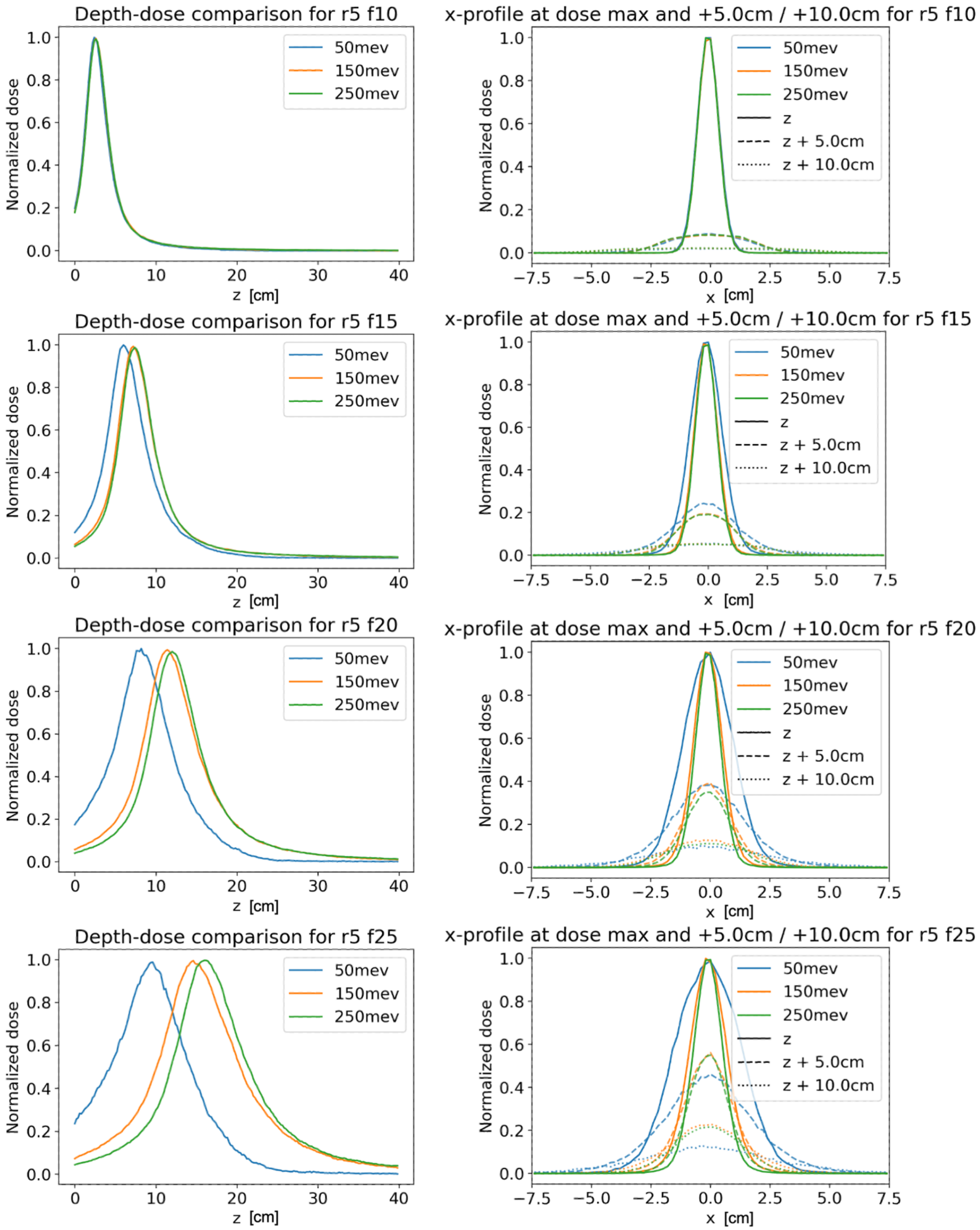

Figure S4: Depth-dose curves at the central axis (left column) for idealized fHEE beams in water with a magnetic lens of radius 5 (r5) placed 7 cm before the water box, with different focal lengths (f10, f15, f20, f25) in the different rows, and different energies 50 MeV (blue), 150 MeV (orange), and 250 MeV (green). Lateral profiles along x (right column) at the dose maximum depth and 5cm, respectively 10 cm, behind the according dose maximum.

Lung case 1 (L1)

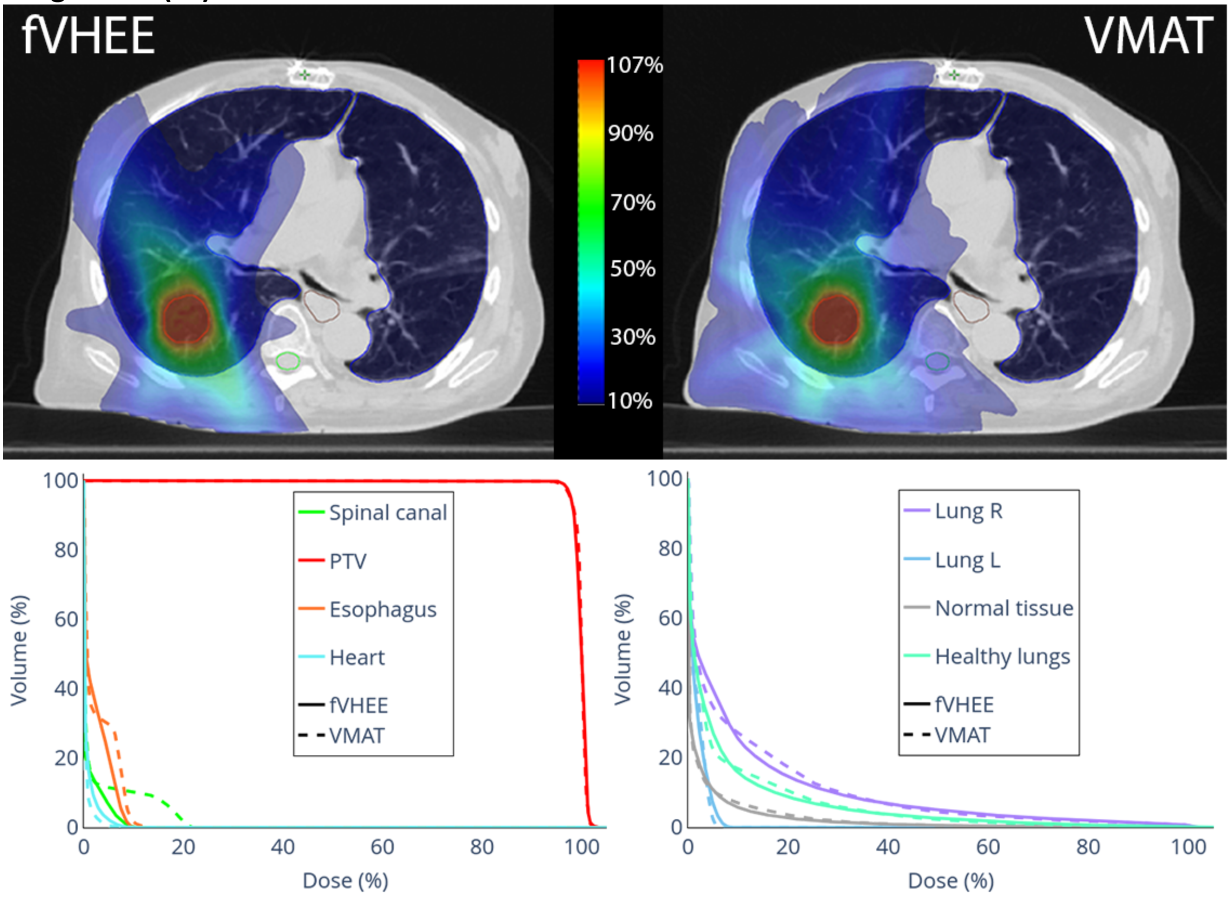

Figure S5: Dose distributions with DVHs for the lung case 1 (L1).

Head and neck case (H)

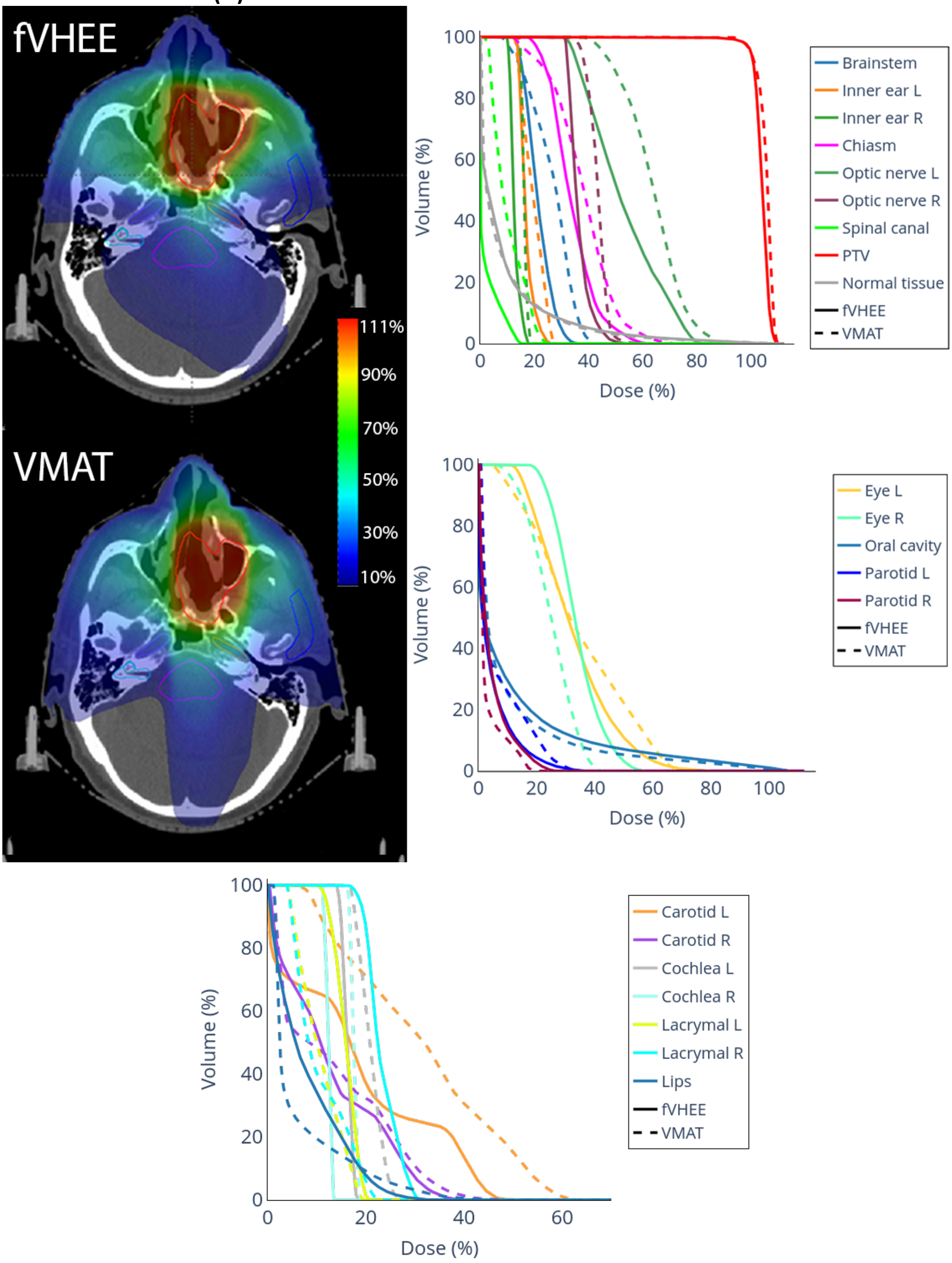

Figure S6: Dose distributions with DVHs for the head and neck case (H).

Prostate case 1 (P1)

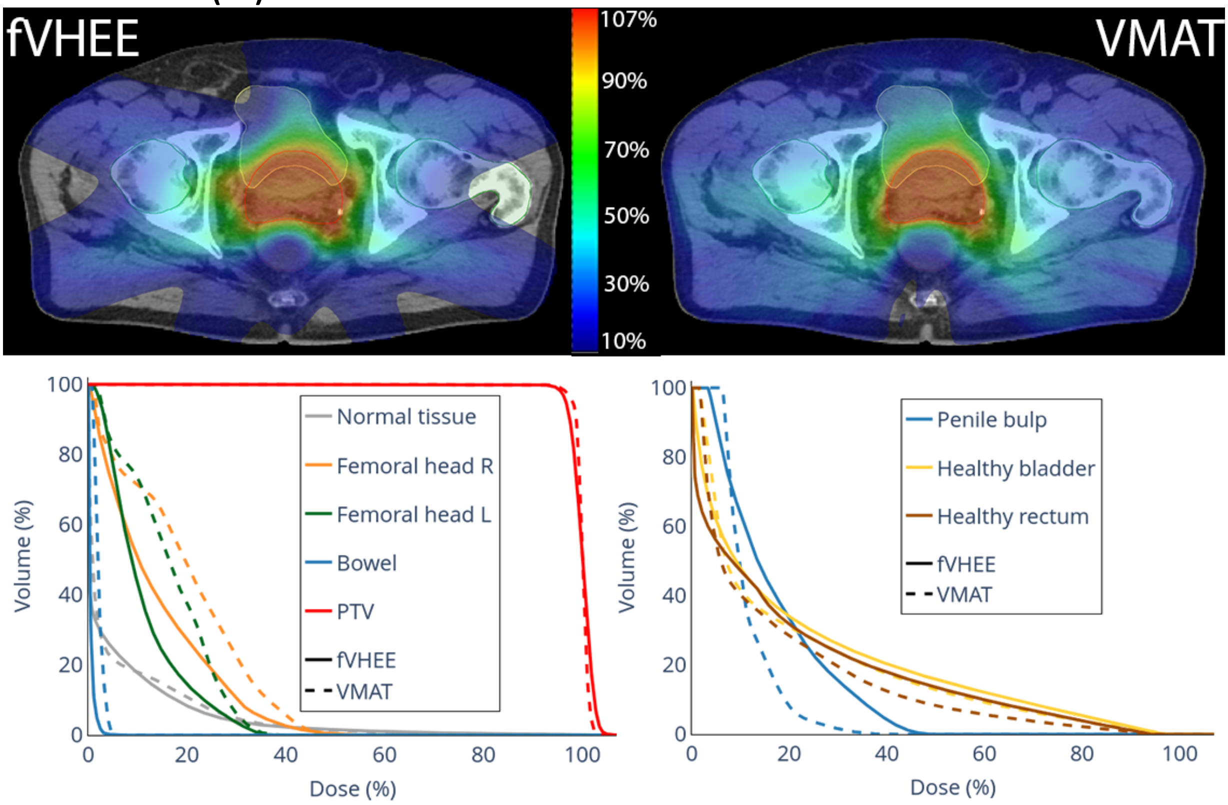

Figure S7: Dose distributions with DVHs for the prostate case 1 (P1).

Prostate case 2 (P2)

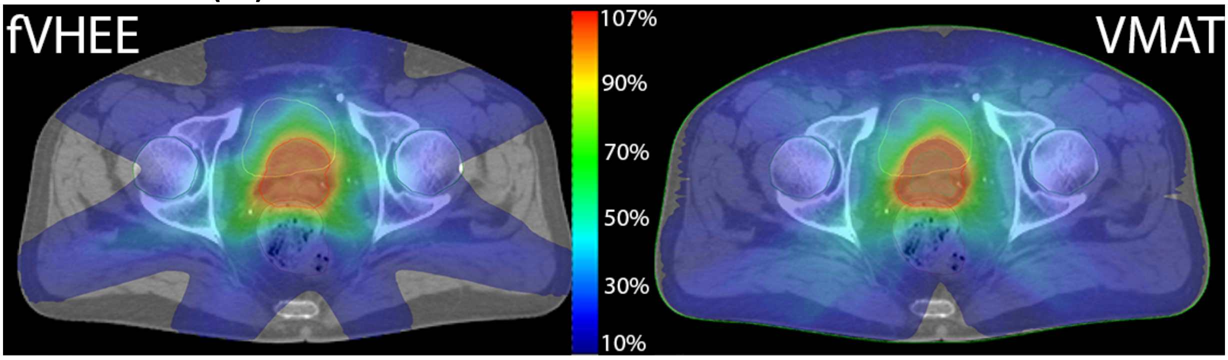

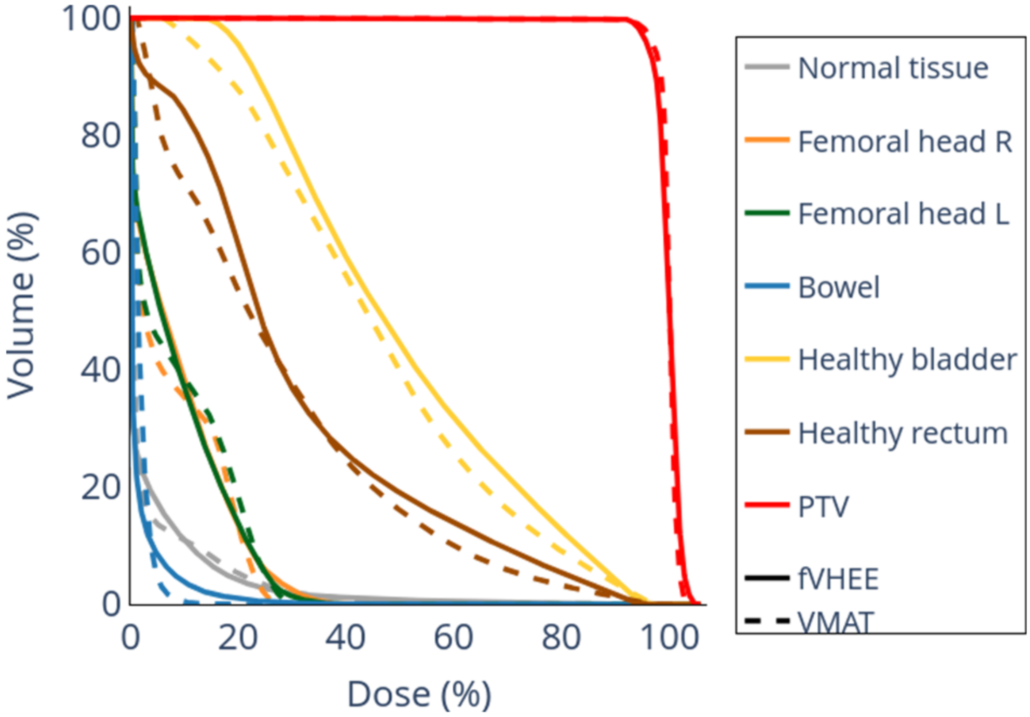

Figure S8: Dose distribution with DVHs for the prostate case 2 (P2).

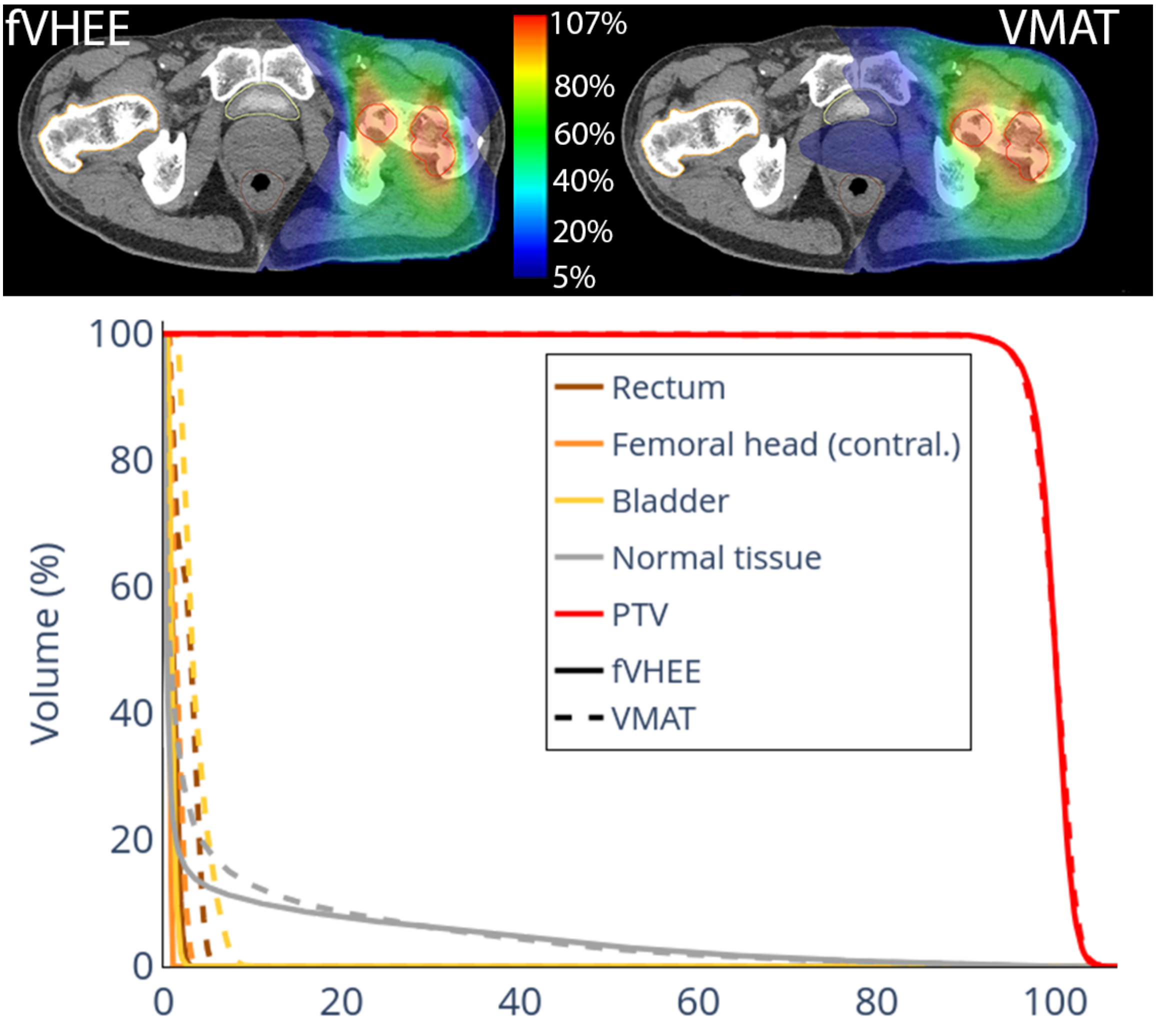

Figure S9: Dose distributions of fVHEE and the clinical VMAT in a representative transversal slice with DVHs for the femoral head case F.

*Table S1: DVH parameters for the fVHEE, the clinical VMAT (Eclipse), and the optimizer-matched VMAT (in-house) plans and their differences for the B case. For each metric, the best value is shown in **bold**.*

Brain (B)

Targets/OARs	Param.	fVHEE	clinical VMAT (cVMAT)	optimizer-matched VMAT (oVMAT)	Diff. fVHEE-cVMAT	Diff. fVHEE-oVMAT	Diff. oVMAT - cVMAT
PTV	$D_{2\%}$ [Gy]	13.0	13.8	**12.9**	-0.8	0.1	-0.9
PTV	D_{mean} [Gy]	12.4	12.8	12.4	-0.4	0.0	-0.4
PTV	$D_{98\%}$ [Gy]	**11.8**	**11.8**	**11.8**	0.0	0.0	0.0
PTV	$V_{95\%}$ [%]	99.5	**99.7**	**99.7**	0.0	0.2	0.2
Eye L	$D_{2\%}$ [Gy]	**0.1**	0.2	0.2	-0.1	-0.1	0.0
Eye L	D_{mean} [Gy]	**0.0**	0.1	0.1	-0.1	-0.1	0.0
Eye R	$D_{2\%}$ [Gy]	**0.0**	0.4	0.7	-0.3	-0.6	0.3
Eye R	D_{mean} [Gy]	**0.0**	0.2	0.4	-0.2	-0.4	0.2
OpticN_L	$D_{2\%}$ [Gy]	**0.1**	0.2	0.2	-0.1	-0.2	0.1
OpticN_L	D_{mean} [Gy]	**0.0**	0.1	0.2	-0.1	-0.1	0.0
OpticN_R	$D_{2\%}$ [Gy]	**0.0**	0.4	0.5	-0.4	-0.5	0.1
OpticN_R	D_{mean} [Gy]	**0.0**	0.3	0.4	-0.3	-0.4	0.2
Chiasm	$D_{2\%}$ [Gy]	**0.2**	0.4	0.4	-0.2	-0.2	0.0
Chiasm	D_{mean} [Gy]	**0.1**	0.3	0.3	-0.2	-0.2	0.0
Lens L	$D_{2\%}$ [Gy]	**0.0**	0.1	0.1	-0.1	-0.1	0.0
Lens L	D_{mean} [Gy]	**0.0**	0.1	0.1	0.0	-0.1	0.0
Lens R	$D_{2\%}$ [Gy]	**0.0**	0.2	0.4	-0.2	-0.4	0.2
Lens R	D_{mean} [Gy]	**0.0**	0.2	0.4	-0.2	-0.4	0.2
Brainstem	$D_{2\%}$ [Gy]	0.9	**0.8**	0.9	0.2	0.0	0.2
Brainstem	D_{mean} [Gy]	**0.3**	0.4	0.4	-0.1	-0.2	0.1
Brain	D2% [Gy]	**12.3**	12.6	**12.3**	-0.3	0.0	-0.3
Brain	D_{mean} [Gy]	**1.6**	1.7	1.7	-0.1	-0.1	0.0
Pituitary gland	$D_{2\%}$ [Gy]	**0.1**	0.2	0.3	-0.2	-0.3	0.1
Pituitary gland	D_{mean} [Gy]	**0.1**	0.2	0.2	-0.2	-0.2	0.1
Cochlea R	$D_{2\%}$ [Gy]	0.7	0.9	**0.6**	-0.2	0.1	-0.3
Cochlea R	D_{mean} [Gy]	**0.4**	**0.4**	**0.4**	0.0	-0.1	0.1
Cochlea L	$D_{2\%}$ [Gy]	0.6	**0.2**	0.3	0.4	0.3	0.1
Cochlea L	D_{mean} [Gy]	0.5	**0.1**	0.2	0.3	0.2	0.1
Normal tissue	D_{mean} [Gy]	**0.6**	**0.6**	**0.6**	0.0	0.0	0.0

*Table S2: DVH parameters for the fVHEE, the clinical VMAT (Eclipse), and the optimizer-matched VMAT (in-house) plans and their differences for the H case. For each metric, the best value is shown in **bold**.*

Head and neck case (H)

Targets/OARs	Param.	fVHEE	clinical VMAT (cVMAT)	optimizer-matched VMAT (oVMAT)	Diff. fVHEE-cVMAT	Diff. fVHEE-oVMAT	Diff. oVMAT - cVMAT
PTV	$D_{2\%}$ [Gy]	54.9	**54.7**	55.7	0.2	-0.7	1.0
PTV	D_{mean} [Gy]	52.0	52.8	52.6	-0.8	-0.6	-0.2
PTV	$D_{98\%}$ [Gy]	48.4	**48.7**	**48.7**	-0.3	-0.3	0.0
PTV	$V_{95\%}$ [%]	98.7	**99.1**	99.0	-0.4	-0.2	-0.2
Carotid L	D2% [Gy]	**23.1**	29.5	24.5	-6.4	-1.4	-5.0
Carotid_L	D_{mean} [Gy]	**9.2**	15.9	13.9	-6.7	-4.7	-2.0
Carotid R	$D_{2\%}$ [Gy]	**17.5**	18.9	20.5	-1.4	-3.1	1.6
Carotid R	D_{mean} [Gy]	**6.5**	**6.6**	8.3	-0.2	-1.8	1.7
Cochlea L	$D_{2\%}$ [Gy]	9.1	14.0	**7.3**	-4.9	1.8	-6.7
Cochlea L	D_{mean} [Gy]	8.1	10.5	**6.5**	-2.4	1.6	-4.0
Cochlea R	$D_{2\%}$ [Gy]	**6.6**	9.7	7.0	-3.1	-0.4	-2.7
Cochlea R	D_{mean} [Gy]	6.2	8.8	**6.2**	-2.6	0.0	-2.6
Chiasm	$D_{2\%}$ [Gy]	29.5	32.7	**26.1**	-3.2	3.4	-6.6
Chiasm	D_{mean} [Gy]	17.2	18.8	**16.1**	-1.7	1.1	-2.8
Eye L	$D_{2\%}$ [Gy]	32.4	33.0	**29.7**	-0.7	2.6	-3.3
Eye L	D_{mean} [Gy]	16.3	17.1	**15.0**	-0.8	1.3	-2.0
Eye R	$D_{2\%}$ [Gy]	25.8	**19.6**	24.1	6.2	1.8	4.5
Eye R	D_{mean} [Gy]	16.9	**12.4**	13.5	4.5	3.3	1.2
Inner ear L	$D_{2\%}$ [Gy]	12.8	14.0	**12.5**	-1.2	0.3	-1.5
Inner ear L	D_{mean} [Gy]	8.6	9.8	**7.3**	-1.3	1.2	-2.5
Inner ear R	$D_{2\%}$ [Gy]	**8.8**	9.2	12.4	-0.4	-3.6	3.3
Innear ear R	D_{mean} [Gy]	**6.4**	8.1	7.5	-1.7	-1.1	-0.6
Lacrymal L	$D_{2\%}$ [Gy]	10.6	**9.6**	13.1	1.1	-2.5	3.5
Lacrymal L	D_{mean} [Gy]	7.9	**5.2**	6.6	2.7	1.3	1.3
Lacrymal R	$D_{2\%}$ [Gy]	15.2	10.7	**9.1**	4.4	6.1	-1.7
Lacrymal R	D_{mean} [Gy]	11.4	**5.2**	5.4	6.2	6.0	0.2
Lips	$D_{2\%}$ [Gy]	**13.0**	17.5	17.7	-4.5	-4.7	0.2
Lips	D_{mean} [Gy]	4.1	**3.3**	3.5	0.8	0.7	0.2
Oral cavity	$D_{2\%}$ [Gy]	47.0	44.5	**43.9**	2.4	3.1	-0.6
Oral cavity	D_{mean} [Gy]	5.9	**5.4**	6.7	0.5	-0.7	1.3
OpticN L	$D_{2\%}$ [Gy]	40.0	45.4	**37.7**	-5.3	2.4	-7.7
OpticN L	D_{mean} [Gy]	26.6	32.1	**26.4**	-5.5	0.3	-5.8
OpticN R	$D_{2\%}$ [Gy]	24.4	26.1	**21.2**	-1.8	3.1	-4.9
OpticN R	D_{mean} [Gy]	18.2	21.7	**16.4**	-3.5	1.8	-5.3
Spinal cord	$D_{2\%}$ [Gy]	**6.7**	11.4	13.5	-4.7	-6.7	2.1
Spinal cord	D_{mean} [Gy]	**1.0**	5.0	8.2	-4.0	-7.1	3.2
Parotid L	$D_{2\%}$ [Gy]	**12.7**	14.3	15.4	-1.6	-2.7	1.1
Parotid L	D_{mean} [Gy]	**2.3**	3.4	2.7	-1.2	-0.5	-0.7
Parotid R	$D_{2\%}$ [Gy]	10.6	**8.2**	11.7	2.4	-1.1	3.5
Parotid R	D_{mean} [Gy]	2.3	**1.6**	2.0	0.7	0.3	0.4
Brainstem	$D_{2\%}$ [Gy]	**16.0**	19.7	17.4	-3.7	-1.4	-2.3
Brainstem	D_{mean} [Gy]	**10.6**	13.2	10.9	-2.6	-0.3	-2.3
Normal tissue	D_{mean} [Gy]	4.4	**4.3**	4.9	0.0	-0.5	0.5

*Table S3: DVH parameters for the fVHEE, the clinical VMAT (Eclipse), and the optimizer-matched VMAT (in-house) plans and their differences for the L1 case. For each metric, the best value is shown in **bold**.*

Lung case 1 (L1)

Targets/OARs	Param.	fVHEE	clinical VMAT (cVMAT)	optimizer-matched VMAT (oVMAT)	Diff. fVHEE-cVMAT	Diff. fVHEE-oVMAT	Diff. oVMAT - cVMAT
PTV	$D_{2\%}$ [Gy]	61.6	**61.2**	62.2	0.4	-0.6	1.0
PTV	D_{mean} [Gy]	59.9	60.0	60.0	-0.1	-0.1	0.0
PTV	$D_{98\%}$ [Gy]	**57.4**	**57.4**	56.7	0.1	0.7	-0.7
PTV	$V_{95\%}$ [%]	**98.9**	98.7	97.4	0.2	1.5	-1.2
Heart	$D_{2\%}$ [Gy]	3.2	**1.9**	3.4	1.3	-0.2	1.5
Heart	D_{mean} [Gy]	0.4	**0.3**	0.5	0.1	-0.1	0.2
Esophagus	$D_{2\%}$ [Gy]	5.1	**4.9**	5.3	0.2	-0.2	0.4
Esophagus	D_{mean} [Gy]	**1.2**	1.4	1.4	-0.2	-0.2	0.0
Spinal cord	$D_{2\%}$ [Gy]	**4.6**	11.9	9.6	-7.3	-5.0	-2.3
Spinal cord	D_{mean} [Gy]	**0.5**	1.3	1.2	-0.8	-0.7	-0.1
Lungs-PTV	D_{mean} [Gy]	**3.8**	**3.8**	4.4	-0.1	-0.7	0.6
Lungs-PTV	V20Gy [%]	**4.8**	5.1	6.1	-0.3	-1.3	1.0
Lung L	D_{mean} [Gy]	**1.0**	**1.0**	1.4	0.0	-0.4	0.4
Lung R	D_{mean} [Gy]	**5.9**	6.0	6.7	-0.1	-0.8	0.7
Normal tissue	D_{mean} [Gy]	**1.3**	1.5	1.8	-0.1	-0.4	0.3

Table S4: DVH parameters for the fVHEE, the clinical VMAT (Eclipse), and the optimizer-matched VMAT (in-house) plans and their differences for the L2 case. For each metric, the best value is shown in ***bold****.*

Lung case 2 (L2)

**Targets/OARs**	**Param.**	**fVHEE**	**clinical VMAT (cVMAT)**	**optimizer-matched VMAT (oVMAT)**	**Diff. fVHEE-cVMAT**	**Diff. fVHEE-oVMAT**	**Diff. oVMAT - cVMAT**
PTV	$D_{2\%}$ [Gy]	62.6	**62.4**	62.9	0.2	-0.3	0.4
PTV	D_{mean} [Gy]	60.0	59.8	60.0	0.2	0.0	0.2
PTV	$D_{98\%}$ [Gy]	**56.9**	55.3	56.2	1.5	0.7	0.8
PTV	$V_{95\%}$ [%]	**97.7**	93.4	96.6	4.4	1.2	3.2
Heart	$D_{2\%}$ [Gy]	**36.7**	38.2	38.8	-1.6	-2.1	0.5
Heart	D_{mean} [Gy]	**5.9**	8.5	8.5	-2.7	-2.6	-0.1
Esophagus	$D_{2\%}$ [Gy]	23.8	29.1	**19.4**	-5.3	4.4	-9.7
Esophagus	D_{mean} [Gy]	**5.4**	7.6	6.8	-2.2	-1.4	-0.8
Spinal cord	$D_{2\%}$ [Gy]	16.2	23.6	**15.2**	-7.4	1.0	-8.4
Spinal cord	D_{mean} [Gy]	**3.1**	5.2	3.6	-2.1	-0.5	-1.6
Lungs-PTV	D_{mean} [Gy]	**6.9**	7.1	7.0	-0.2	-0.1	-0.1
Lungs-PTV	V20Gy [%]	9.2	**4.1**	6.6	5.1	2.6	2.5
Lung L	D_{mean} [Gy]	**4.0**	4.8	5.5	-0.8	-1.5	0.7
Lung R	D_{mean} [Gy]	9.8	9.6	**8.8**	0.3	1.0	-0.8
Liver	$D_{2\%}$ [Gy]	16.9	**11.5**	14.1	5.3	2.8	2.6
Liver	D_{mean} [Gy]	2.0	**1.5**	1.8	0.5	0.2	0.3
Trachea	$D_{2\%}$ [Gy]	26.0	32.5	**25.0**	-6.4	1.1	-7.5
Trachea	D_{mean} [Gy]	**7.7**	11.3	9.7	-3.6	-2.0	-1.6
Normal tissue	D_{mean} [Gy]	**2.5**	2.6	2.8	-0.2	-0.3	0.2

*Table S5: DVH parameters for the fVHEE, the clinical VMAT (Eclipse), and the optimizer-matched VMAT (in-house) plans and their differences for the P1 case. For each metric, the best value is shown in **bold**.*

Prostate case 1 (P1)

Targets/OARs	Param.	fVHEE	clinical VMAT (cVMAT)	optimizer-matched VMAT (oVMAT)	Diff. fVHEE-cVMAT	Diff. fVHEE-oVMAT	Diff. oVMAT - cVMAT
PTV	$D_{2\%}$ [Gy]	62.4	**61.7**	62.6	0.7	-0.1	0.8
PTV	D_{mean} [Gy]	60.0	60.1	60.1	-0.1	-0.1	0.0
PTV	$D_{98\%}$ [Gy]	56.8	**58.0**	57.8	-1.2	-1.0	-0.2
PTV	$V_{95\%}$ [%]	97.3	99.1	**99.5**	-1.7	-2.1	0.4
Rectum	$D_{2\%}$ [Gy]	**56.0**	56.5	57.8	-0.4	-1.7	1.3
Rectum	D_{mean} [Gy]	12.1	**10.6**	11.9	1.5	0.2	1.3
Rectum wall	$D_{2\%}$ [Gy]	**57.5**	59.2	59.0	-1.7	-1.5	-0.2
Rectum wall	D_{mean} [Gy]	13.6	**12.0**	13.2	1.6	0.5	1.2
Penile bulb	$D_{2\%}$ [Gy]	28.2	**13.6**	14.5	14.6	13.6	0.9
Penile bulb	D_{mean} [Gy]	10.6	**6.8**	7.0	3.7	3.6	0.2
FemoralHead R	$D_{2\%}$ [Gy]	**25.1**	26.4	25.2	-1.4	-0.2	-1.2
FemoralHead R	D_{mean} [Gy]	**8.1**	11.6	11.8	-3.5	-3.7	0.2
FemoralHead L	$D_{2\%}$ [Gy]	**19.3**	20.0	17.2	-0.7	2.1	-2.8
FemoralHead L	D_{mean} [Gy]	**6.6**	9.7	8.4	-3.1	-1.8	-1.3
Bladder	$D_{2\%}$ [Gy]	60.7	60.4	**59.8**	0.2	0.9	-0.7
Bladder	D_{mean} [Gy]	15.1	**14.1**	14.9	1.0	0.2	0.8
Bladder wall	$D_{2\%}$ [Gy]	61.5	60.7	**60.4**	0.7	1.1	-0.4
Bladder wall	D_{mean} [Gy]	17.3	**17.0**	17.5	0.3	-0.3	0.5
Bowel	$D_{2\%}$ [Gy]	**1.5**	2.6	2.6	-1.1	-1.1	0.0
Bowel	D_{mean} [Gy]	**0.3**	1.3	1.3	-1.0	-1.0	0.1
Normal tissue	D_{mean} [Gy]	**3.2**	3.4	3.6	-0.2	-0.4	0.2

*Table S6: DVH parameters for the fVHEE, the clinical VMAT (Eclipse), and the optimizer-matched VMAT (in-house) plans and their differences for the P2 case. For each metric, the best value is shown in **bold**.*

Prostate case 2 (P2)

Targets/OARs	Param.	fVHEE	clinical VMAT (cVMAT)	optimizer-matched VMAT (oVMAT)	Diff. fVHEE-cVMAT	Diff. fVHEE-oVMAT	Diff. oVMAT - cVMAT
PTV	$D_{2\%}$ [Gy]	62.5	**62.1**	64.3	0.4	-1.8	2.2
PTV	D_{mean} [Gy]	59.9	60.1	60.1	-0.2	-0.2	0.0
PTV	$D_{98\%}$ [Gy]	56.2	**57.3**	56.4	-1.1	-0.2	-0.8
PTV	$V_{95\%}$ [%]	96.5	**98.3**	96.2	-1.9	0.2	-2.1
Rectum	$D_{2\%}$ [Gy]	**57.8**	59.4	59.9	-1.5	-2.0	0.5
Rectum	D_{mean} [Gy]	19.1	**17.3**	18.0	1.8	1.1	0.7
FemoralHead R	$D_{2\%}$ [Gy]	18.5	**14.9**	21.2	3.6	-2.7	6.3
FemoralHead R	D_{mean} [Gy]	5.2	4.7	6.4	0.5	-1.2	1.7
FemoralHead L	$D_{2\%}$ [Gy]	17.1	**16.4**	25.5	0.7	-8.4	9.1
FemoralHead L	D_{mean} [Gy]	**5.1**	5.2	7.8	-0.2	-2.7	2.5
Bladder	$D_{2\%}$ [Gy]	61.7	**61.5**	63.7	0.2	-2.0	2.2
Bladder	D_{mean} [Gy]	37.2	35.4	**34.2**	1.8	3.0	-1.2
Bowel	$D_{2\%}$ [Gy]	8.6	**3.9**	5.7	4.7	2.8	1.8
Bowel	D_{mean} [Gy]	**0.9**	1.1	1.4	-0.2	-0.4	0.2
Normal tissue	D_{mean} [Gy]	**1.9**	2.0	2.4	-0.1	-0.5	0.4

*Table S7: DVH parameters for the fVHEE, the clinical VMAT (Eclipse), and the optimizer-matched VMAT (in-house) plans and their differences for the F case. For each metric, the best value is shown in **bold**. Cells shaded in green indicate metrics where the first mentioned modality outperforms the second mentioned (darker = greater advantage), while cells shaded in red indicate metrics where the first performs worse than the second (darker = greater disadvantage).*

Femoral head case (F1)

Targets/OARs	Param.	fVHEE	clinical VMAT (cVMAT)	optimizer-matched VMAT (oVMAT)	Diff. fVHEE-cVMAT	Diff. fVHEE-oVMAT	Diff. oVMAT - cVMAT
PTV	$D_{2\%}$ [Gy]	48.3	48.3	**48.2**	0.0	0.1	-0.1
PTV	D_{mean} [Gy]	46.4	46.4	46.4	0.0	0.0	0.0
PTV	$D_{98\%}$ [Gy]	**43.5**	43.4	43.4	0.1	0.0	0.0
PTV	$V_{95\%}$ [%]	**96.4**	96.1	96.3	0.3	0.2	0.0
Rectum	$D_{2\%}$ [Gy]	**1.1**	2.4	1.6	-1.3	-0.5	-0.8
Rectum	D_{mean} [Gy]	**0.5**	1.3	1.0	-0.8	-0.5	-0.3
Bladder	$D_{2\%}$ [Gy]	**0.9**	3.6	2.4	-2.6	-1.5	-1.2
Bladder	D_{mean} [Gy]	**0.5**	1.7	1.2	-1.2	-0.8	-0.5
FemoralHead R	$D_{2\%}$ [Gy]	**0.5**	1.4	0.7	-1.0	-0.2	-0.7
FemoralHead R	D_{mean} [Gy]	**0.3**	0.7	0.4	-0.4	-0.1	-0.3
NormalTissue	D_{mean} [Gy]	**2.2**	2.6	2.5	-0.4	-0.3	-0.1

Supplementary material A: Detailed spot placement

The spot placement is performed in the following way: first, an extended bounding box of the PTV was computed:

$(x_{min}, x_{max}, y_{min}, y_{max}, z_{min}, z_{max}) = (min(x_{PTV}) - m, max(x_{PTV}) + m, \; min(y_{PTV}) - m, max(y_{PTV}) + m, min(z_{PTV}) - m, max(z_{PTV}) + m)$
with x_{min}, x_{max}, y_{min}, y_{max}, z_{min},and z_{max}, the bounds of the box, defined by the minimum and maximum coordinate of the PTV in each direction and adding a margin m.

Within this volume, a 3D hexagonal (diamond-pattern) grid of candidate focal spots was generated in steps of 4k. The spacing factor k was chosen empirically to balance homogeneity, conformity and computation time. At each grid origin [x, y, z] eight additional offsets ensured dense, uniform coverage:

(i) $[x, y, z]$;
(ii) $[x, y + 2k, z + 2k]$;
(iii) $[x + 2k, y, z + 2k]$;
(iv) $[x + 2k, y + 2k, z]$;
(v) $[x + k, y + 3k, z + k]$;
(vi) $[x + k, y + k, z + 3k]$;
(vii) $[x + 3k, y + k, z + k]$;
$(viii)$ $[x + 3k, y + 3k, z + 3k]$;

To restrict beamlets to the actual PTV shape while preserving margin coverage, we generated all corner offsets $\boldsymbol{dx}, \boldsymbol{dy}, \boldsymbol{dz} \in \{-\boldsymbol{m}, \boldsymbol{0}, \boldsymbol{m}\}$ for each PTV voxel [x, y, z]. Then, the convex hull from these extended points was created by Delaunay triangulation. Lastly, only grid points lying within this hull were retained, reducing unnecessary beamlet dose calculations.

Supplementary material B: Definition of the objective function and used objectives and weights

The objective function F is defined the same as previously reported by our group (Müller et al., A hybrid column generation and simulated annealing algorithm for direct aperture optimization, 2022). For convenience reason the relevant parts of the objective function for this work are here repeated:

$$F(D_i) = \sum_{r=1}^{N_{DV}} p_{r,DV} \cdot f_{r,DV}(D_i) + \sum_{q=1}^{N_{NT}} p_{q,NT} \cdot f_{q,NT}(D_i)$$

$$f_{r,DV}(D_i) = \frac{1}{V_{str,r}} \sum_{i=1}^{M_r} v_{i,r} \cdot \theta\big(\mathrm{a_r} \cdot (D_i - D_r)\big) \cdot \theta\big(a_r \cdot (D(V_r) - D_i)\big) \cdot (D_i - D_r)^2$$

$$f_{q,NT}(D_i) = \frac{1}{V_{str,q}} \sum_{i=1}^{M_q} v_{i,q} \cdot \theta\big(D_i - D_{i,q}\big) \cdot \big(D_i - D_{i,q}\big)^2$$

$$D_{i,q} = \begin{cases} d_0 e^{-b(x_i - x_{start})} + d_\infty\big(1 - e^{-b(x_i - x_{start})}\big), & if\ x_i \geq x_{start} \\ d_0 & , otherwise \end{cases}$$

The following tables summarizes the given objectives and their priorities:

Table S8: Optimization objectives and priorities used for inverse-optimized planning in the B and H cases. Parameters define the objective function components for target coverage and organ sparing, including distance-dependent fall-off (b) for normal tissue.

Patient Case	Structure	Type	Priority (p)	Dose (D) [%]	Volume (V) [%]	StartDistance (x_start) [cm]	EndDose (d_∞) [%]	FallOff (b)
B	PTV	Upper	1200	102	0			
	PTV	Lower	1200	99	95			
	PTV	Lower	12000	98	100			
	Brainstem	Upper	3820	5	0			
	Brainstem	Upper	920	3	10			
	Brainstem	Upper	720	1	30			
	Brain	Upper	220	40	9			
	Brain	Upper	220	60	5			
	Brain	Upper	220	90	3			
	Eye left	Upper	520	4	0			
	Eye right	Upper	520	4	0			
	Lens left	Upper	520	4	0			
	Lens right	Upper	520	4	0			
	Optic nerve left	Upper	720	4	0			
	Optic nerve right	Upper	720	4	0			
	Chiasm	Upper	820	4	0			
	Pituitary Gland	Upper	820	4	0			
	Chochlea left	Upper	420	4	0			
	Chochlea right	Upper	420	4	0			
	NormalTissue	NT	1000	2	90	0.5	40	0.2
H	PTV	Upper	5200	102	0.1			
	PTV	Upper	15200	105	0			
	PTV	Upper	252000	106	0			
	PTV	Lower	15200	99	95			
	PTV	Lower	19200	98	100			
	BrainStem	Upper	820	35	0			
	BrainStem	Upper	420	20	10			
	BrainStem	Upper	420	15	30			
	Cochlea_L	Upper	120	10	0			
	Cochlea_R	Upper	220	10	0			
	Carotid_R	Upper	220	30	0			
	Carotid_L	Upper	120	35	0			
	Eye_L	Upper	320	50	1			
	Eye_R	Upper	320	40	1			
	Parotid_L	Upper	920	5	5			
	Parotid_R	Upper	920	6	6			
	OralCavity	Upper	320	15	15			
	OralCavity	Upper	420	60	3			
	SpinalCord	Upper	320	15	0			
	OpticN_L	Upper	220	50	0			
	OpticN_R	Upper	220	30	0			
	OpticChiasm	Upper	420	33	0			
	NormalTissue	Upper	152000	104	0			
	NormalTissue	NT	800	2	90	0.5	40	0.2

Table S9: Optimization objectives and priorities used for inverse-optimized planning in the L1, L2, and P1 cases. Parameters define the objective function components for target coverage and organ sparing, including distance-dependent fall-off (b) for normal tissue.

Patient Case	Structure	Type	Priority (p)	Dose (D) [%]	Volume (V) [%]	StartDistance (x_start) [cm]	EndDose (d_∞) [%]	FallOff (b)
L1	PTV	Upper	3300	101	0			
	PTV	Lower	3800	99	95			
	PTV	Lower	3800	97	100			
	Lung_R	Upper	400	5	25			
	Lung_R	Upper	600	60	2			
	Lung_R	Upper	550	90	1			
	Lung_Healthy	Upper	550	5	20			
	Heart	Upper	500	1	1			
	Esophagus	Upper	400	5	1			
	SpinalCanal	Upper	400	10	1			
	NormalTissue	NT	600	40	90	0.5	40	0.2
L2	PTV	Upper	72300	104	0			
	PTV	Lower	7600	99	95			
	PTV	Lower	72000	97	100			
	Lung_Healthy	Upper	5100	5	20			
	Lung_R	Upper	2100	5	25			
	Lung_R	Upper	2300	60	2			
	Lung_R	Upper	2100	90	0			
	Heart	Upper	600	50	0			
	Heart	Upper	1200	10	10			
	Esophagus	Upper	3000	25	0			
	SpinalCanal	Upper	3850	20	0			
	Liver	Upper	500	5	5			
	Liver	Upper	3700	35	0			
	Trachea	Upper	100	10	30			
	Trachea	Upper	300	17	17			
	Trachea	Upper	3750	40	0			
	NormalTissue	Upper	700	108	0			
	NormalTissue	NT	600	2	90	0.5	40	0.2
P1	PTV	Upper	1999	102	0			
	PTV	Upper	79999	107	0			
	PTV	Lower	1999	99	95			
	PTV	Lower	39999	97	100			
	Bladder	Upper	1999	95	0			
	Bladder	Upper	1999	80	10			
	Bladder	Upper	199	30	25			
	BladderWall	Upper	1999	80	0			
	PenileBulb	Upper	2999	30	0			
	PenileBulb	Upper	199	10	20			
	FemoralHead_L	Upper	19	40	0			
	FemoralHead_R	Upper	19	50	0			
	Bowel	Upper	19	12	0			
	Rectum	Upper	1999	95	0			
	Rectum	Upper	1999	40	15			
	RectumWall	Upper	199	90	0			
	NormalTissue	Nt	199	20	90	0.5	40	0.2

Table S10: Optimization objectives and priorities used for inverse-optimized planning in the P2 and F cases. Parameters define the objective function components for target coverage and organ sparing, including distance-dependent fall-off (b) for normal tissue.

**Patient Case**	**Structure**	**Type**	**Priority (p)**	**Dose (D) [%]**	**Volume (V) [%]**	**StartDistance (x_start) [cm]**	**EndDose (d_∞) [%]**	**FallOff (b)**
P2	PTV	Upper	5300	101	0			
	PTV	Lower	8000	107	0			
	PTV	Lower	9800	99	95			
	PTV	Upper	18000	97	100			
	Bladder	Upper	700	95	0			
	Bladder	Upper	1000	40	55			
	Bladder	Upper	600	64	30			
	FemoralHead_L	Upper	450	22	0			
	FemoralHead_R	Upper	450	22	0			
	Bowel	Upper	400	15	0			
	Bowel	Upper	9000	20	0			
	Rectum	Upper	700	80	0			
	Rectum	Upper	1000	29	40			
	Rectum	Upper	1500	60	10			
	NormalTissue	NT	1600	40	90	0.5	40	0.2
F	PTV	Upper	3300	101	0			
	PTV	Lower	3800	99	95			
	PTV	Lower	3800	97	100			
	Rectum	Upper	6900	1	0			
	FemoralHead_R	Upper	3200	1	0			
	Bladder	Upper	6900	1	0			
	NormalTissue	NT	1600	30	90	0.5	40	0.2

Supplementary material C

Table S11: RATING table according to Hansen et al. [43].

	RATING score sheet	Points	Applicable/ relevant	Answer yes
**Questions for the Introduction**				
	The study aim formulated by research questions			
1	Does the study have a concise and precise study aim, defined with a restricted number of interconnected questions?	10		☑
	The motivation for the research questions			
2	Has relevant up to date literature been included to support the need for the current study?	5		☑
3	Does the study address an existing knowledge gap?	10		☑
**Questions for Materials and Methods**				
4	Is the global study design adequate for answering the posed research questions?	10		☑
5	Is the global study design described in sufficient detail for others to interpret and reproduce the results?	5		☑
	Patient cohort			
6	Are the inclusion and exclusion criteria of the patient cohort described?	1	☐	☐
7	Is the clinical patient information of the cohort presented, including disease type, site(s) and clinical staging?	1	☐	☐
8	Is the included number of patients stated, explained and justified?	1	☑	☑
9	Has there been consideration of the need for ethical and/or legal approval for the study and if needed, is there a statement about this?	5		☑
	Imaging procedures			
10	Have the scanning parameters been reported in sufficient detail (image modalities, equipment model, slice thickness, voxel size, patient position (e.g. head first, supine, etc.) etc.)?	1	☐	☐
11	Has the applied immobilisation equipment been described, (e.g. vendor and type, standard settings, etc.) where relevant?	1	☐	☐
	Treatment machine and settings			
12	Have the treatment machine and relevant parameters been described with sufficient detail (model, beam energy, MLC, etc.)?	1	☐	☐
13	Have the monitor unit reference conditions been defined, where relevant?	1	☐	☐
	Definition of targets and OARs			
14	Has GTV definition been described in sufficient detail, with references if possible?	1	☐	☐
15	Has CTV definition been described in sufficient detail, with references if possible?	1	☐	☐
16	Has the establishment of PTVs (or alternatively robustness settings) been described in sufficient detail?	1	☐	☐
17	Have PTV sizes in the patient cohort been described?	1	☑	☑
18	Have OAR definitions been described in sufficient detail, with references if possible?	1	☐	☐
19	Have PRV margins been described in sufficient detail, with references if available?	1	☐	☐

	Treatment planning system and dose calculation			
20	Have all applied dose calculation algorithms been described in sufficient detail?	1	☑	☑
21	For any commercial software used, have the manufacturer, algorithms and specific versions been stated?	1	☑	☑
22	Have all relevant user parameters and settings in the TPS been reported, e.g. beams, dose grid, control point spacing?	1	☑	☑
23	Have all volumes been evaluated with the same software/methodology?	1	☑	☑
	Planning aims and optimisation			
24	Are clear planning aims defined, including imposed hard constraints and planning objectives (with or without soft constraints)?	5		☐
25	Has the ranking of planning objectives (priorities) been described?	5		☐
26	Is the dose prescription clearly defined?	10		☑
27	Is there a narrative description of the applied optimisation process, including the handling of all objectives with their ranking?	5		☐
28	If manual intervention during or after optimisation is allowed, has this been described?	1	☑	☐
	Bias mitigation			
29	Have enough study details been provided such that bias issues could be noted?	5		☑
30	Has bias been sufficiently mitigated to reliably answer the posed research question?	10		☑
	Plan acceptability – minor and major protocol deviations			
31	Was the procedure for assessment of plan acceptability well-described?	1	☐	☐
32	Was the procedure for assessment of minor and major protocol deviations well described?	1	☐	☐
	Plan (re-)normalisation for plan comparisons			
33	Has plan (re-)normalisation been described sufficiently?	1	☑	☑
	Dose-volume parameters for plan evaluation and comparison			
34	Have sufficiently comprehensive dose-volume parameters been used for plan evaluations and comparisons?	5		☑
	Population-mean DVHs			
35	Has the algorithm for creating population-mean/median DVHs been reported?	1	☐	☐
36	Have the definitions of confidence intervals been included?	1	☐	☐
	Plan evaluations by clinicians			
37	Have clinicians scored plans to assess quality?	1	☐	☐
38	Were plan comparisons by clinicians blinded?	1	☐	☐
	Predicted tumour control probability and normal tissue complication probabilities for plan evaluation and comparison			
39	Have any applied TCP models been described and referenced?	1	☐	☐
40	Have any applied NTCP models been described and referenced?	1	☐	☐
	Plan deliverability and complexity			
41	Have methods used to assess plan deliverability and complexity been described in sufficient detail?	1	☐	☐
	Composite plan quality metrics			
42	Is there a sufficient basis (e.g. in the literature) for any selected composite plan quality metrics?	1	☐	☐
43	Is there an adequate description of the calculation of the composite plan quality metrics?	1	☐	☐
	Planning and delivery times			
44	Has measurement of planning times been described in sufficient detail?	1	☐	☐
45	Has the establishment of delivery times been described in sufficient detail?	1	☐	☐
	Statistical analysis			
46	Have proper statistical methods been used and described in sufficient detail?	5		☐
47	In case of multiple testing for research questions, has this been handled appropriately?	1	☐	☐

**Questions for Results**				
48	Does the provided data contribute to (at least partly) answering all aspects of the research questions, e.g. plan acceptability, dosimetric quality, deliverability and planning and delivery times?	10		☑
	Dose distribution reporting			
49	Are complete summaries of the dose distributions in the patient cohort provided (low doses, high doses, OARs, PTV, patient, etc.)?	5		☑
50	Are tables and figures optimised to clearly present the results obtained?	1	☑	☑
51	Have the answers to the research questions been illustrated for an example patient by providing dose distributions, DVHs, etc.?	1	☑	☑
	Plan acceptability reporting – minor and major protocol deviations			
52	In case of treatment technique or planning technique comparisons, was plan acceptability reported separately for each technique?	1	☑	☑
53	Has plan acceptability been reported in sufficient detail: how many plans were acceptable, how many were not and for what reasons (e.g. violation of hard constraints, violation of soft constraints, other reasons)?	1	☐	☐
54	Was there adequate reporting of minor and major protocol deviations?	1	☐	☐
	Deliverability and complexity reporting			
55	Has the deliverability of the plans been adequately reported?	1	☑	☑
56	Have plan deliverability and complexity been investigated in sufficient detail in relation to the posed research questions?	1	☐	☐
	Planning and delivery times reporting			
57	Have planning and delivery times been adequately evaluated and reported?	1	☐	☐
	Patient-specific analyses reporting			
58	Is there sufficient description of inter-patient variations in the results presented?	1	☐	☐
59	Have outlier patients been reported and has any exclusion from population analyses been sufficiently motivated and explained?	1	☐	☐
	Statistical reporting			
60	Are the p-values reported appropriately?	1	☐	☐
61	Are there confidence intervals for the appropriate parameters?	1	☐	☐
**Questions for discussions**				
62	Is there an overall interpretation of the data presented in the Results section as to how the posed research questions are answered?	10		☑
	Comparison with literature			
63	Has the study been sufficiently discussed in the context of existing literature?	5		☑
	Clinical and statistical significance			
64	Does the discussion focus on statistically significant results?	1	☐	☐
65	Is the potential clinical significance of the results clearly discussed (assuming practical application would be feasible)?	5		☑
	Clinical applicability of the study			
66	Is future the clinical applicability sufficiently discussed?	1	☑	☑
	Study limitations			
67	Has the impact of the study limitations on the provided answers to the research questions been sufficiently discussed?	10		☑
	Future work			
68	Has the potential future work arising from the study been discussed?	1	☑	☑

**Questions for conclusions**				
69	Do the presented conclusions represent answers to the posed research questions?	5		☑
70	Are the conclusions fully supported by the results?	5		☑
71	Are the conclusions a fair summary of all results?	5		☑
**Questions for supplementary**				
	Supplementary materials			
72	Is the information presented in the supplementary material of sufficient relevance?	1	☑	☑
73	Is the presentation of the included information of sufficient quality, including readability?	1	☑	☑
74	Has sufficient underlying data been made available or a willingness to share data been indicated, within local data sharing restrictions?	5		☐
**RATING remarks**				
75	Is the RATING score added to the manuscript?	5		☑
76	Is the accompanying question table added to the cover letter or the supplementary material?	1	☑	☑
	RATING score	86%		
	**RATING fraction**	156	of	182
